# Ultrasound-based assessment of peri-implant mucosal thickness: an ex vivo comparative study with artificial intelligence-assisted image analysis

**DOI:** 10.1186/s12903-026-08665-0

**Published:** 2026-06-27

**Authors:** Fabian Christleven, Peter Broessner, Nikol Petrova, Stefan Wolfart, Klaus Radermacher, Juliana Marotti

**Affiliations:** 1 Division of Restorative Dentistry, Periodontology and Prosthodontics, Department of Dental Medicine and Oral Health, Medical University Graz, Graz, 8010 Austria; 2 Department of Prosthodontics and Biomaterials, Center for Implantology, Medical School RWTH Aachen University, Aachen, 52074 Germany; 3 Chair of Medical Engineering, Helmholtz Institute for Biomedical Engineering, RWTH Aachen University, Aachen, 52074 Germany

**Keywords:** Ultrasonography, Periodontics, Cone-beam computed tomography, Mucosa, Oral diagnosis, Artificial intelligence

## Abstract

**Background:**

Mucosal thickness (MT) is a key factor influencing peri-implant soft-tissue response and outcomes in dental implantology. This ex vivo study evaluated the agreement of peri-implant MT measurements obtained using ultrasound (US) standardized with a custom probe holder, compared with transgingival probing (TP) and cone-beam computed tomography (CBCT).

**Methods:**

Porcine hemimandibles (*n* = 18) underwent guided implant placement. MT was measured at five standardized points using four approaches: (1) US with expert annotation, (2) US with artificial intelligence (AI)-based image segmentation, (3) CBCT, and (4) TP. US images (18 MHz) were independently annotated by two trained specialists; a deep-learning-based method was used to derive automated MT measurements. Method differences were analyzed using a linear mixed-effects model; agreement was assessed using intraclass correlation coefficients (ICCs) and Bland–Altman analysis.

**Results:**

The overall method effect was not significant (*p* = 0.105). Pairwise comparisons showed no significant difference between expert-annotated US and TP (*p* = 0.328), whereas CBCT yielded higher MT values than TP (*p* = 0.035). Agreement was moderate for expert-annotated US versus TP (ICC = 0.58; 95% confidence interval (CI): 0.42–0.70) and for expert-annotated US versus AI-segmented US (ICC = 0.67; 95% CI: 0.53–0.77), but poor for expert-annotated US versus CBCT (ICC = 0.14; 95% CI: −0.05–0.33). Bland–Altman analysis showed mean differences (95% limits of agreement) of 0.08 mm (− 0.96 mm to + 1.13 mm) for expert-annotated US − TP, − 0.01 mm (− 0.68 mm to + 0.67 mm) for expert-annotated US−AI-segmented US, and − 0.30 mm (− 2.29 mm to + 1.68 mm) for expert-annotated US−CBCT.

**Conclusions:**

Under controlled ex vivo conditions, expert-annotated US standardized with a custom probe holder showed moderate comparative agreement with TP, while AI-segmented measurements showed moderate agreement with expert annotation. CBCT showed limited agreement with US. This integrated approach represents a proof-of-concept requiring further in vivo validation.

**Supplementary Information:**

The online version contains supplementary material available at 10.1186/s12903-026-08665-0.

## Background

Many dental procedures, such as implant placement, periodontal surgery, root coverage, and orthodontic treatment, require a precise assessment of mucosal thickness (MT) to predict soft-tissue behavior and minimize postoperative complications [1–7]. Recent data suggest that MT is associated with peri-implant health status and mucosal recession [8]. MT has also been proposed as a parameter to differentiate between healthy and diseased peri-implant sites, as soft-tissue thickness may change under inflammatory conditions [9]. Although transgingival probing (TP) remains a commonly used method for MT measurement, it is invasive, operator-dependent, and prone to errors from tissue compression and angulation [10–15]. Cone-beam computed tomography (CBCT) has been proposed as an alternative, providing three-dimensional imaging of hard and soft tissues [16–20]. However, limited soft-tissue contrast, beam-hardening artifacts and radiation exposure restrict its clinical reliability for assessing MT [21–23].

Ultrasound (US) has emerged as a promising modality for non-invasive tissue evaluation in dentistry [24–30]. With spatial resolution sufficient for accurate MT measurements and the ability to provide real-time assessment of keratinized tissue characteristics and inflammatory changes [31–35], including peri-implantitis [36–40], US offers notable advantages across multiple stages of implant therapy [41–43]. However, the absence of standardized acquisition protocols [44] and the challenge of ensuring stable probe positioning in the confined oral environment have limited its widespread application. To date, most in vivo US studies have relied on freehand probe placement, which may yield inconsistent measurements due to variations in probe angulation and applied pressure.

A further challenge lies in the interpretation of US images as most dentists have little or no experience with sonography. Sonographic image interpretation is not yet a standard component of dental training, and diagnostic proficiency is therefore often confined to clinicians and researchers with specific expertise [45]. Integration of artificial intelligence (AI)-based segmentation may reduce this barrier by facilitating standardized US-based MT assessment and improving accessibility for dentists irrespective of their prior ultrasound experience [46]. Recent deep learning approaches have shown promise for segmenting mucosal, dental, and bony structures from US images, potentially enhancing diagnostic reliability and reducing operator dependence [46, 47].

To address these limitations, a customized US probe holder that integrates into a surgical guide has been developed (German patent application, DE 10 2024 113 730 A1; see Additional file 1). This system stabilizes the probe during peri-implant measurements and reduces positional variability. In addition, an AI-based segmentation tool for automated MT analysis in US images has been implemented.

This ex vivo study on porcine mandibles aimed to evaluate the agreement of MT measurements obtained via US using a customized probe holder combined with expert image annotation and AI-based image segmentation. US measurements were compared with TP and CBCT. We hypothesized that US would show close agreement with TP and greater comparative agreement relative to CBCT.

## Methods

### Samples, inclusion and exclusion criteria

This study did not involve live animals or procedures regulated under Directive 2010/63/EU of the European Parliament. All specimens were obtained post-slaughter from a local butcher, and all experimental procedures were performed at the Department of Prosthodontics and Biomaterials, Medical School RWTH Aachen University in accordance with EU guidelines for the ethical use of animal-derived materials. Therefore, approval from an animal ethics committee was not required.

A total of 24 porcine hemimandibles were obtained in the period from November 2023 to July 2025. Specimens were screened in multiple phases based on predefined criteria. Inclusion criteria were defined as: (1) freshly slaughtered juvenile porcine hemimandibles with intact bone at the planned implant site between the first premolar and canine, (2) complete mucosal coverage with no laceration in the region of the attached gingiva, and (3) the presence of sufficient stable teeth to ensure reliable fixation of the surgical guide.

Exclusion criteria were: (1) pre-existing macroscopic bone defects or partial detachment of the attached gingiva, or (2) a missing canine, or fractured/unstable teeth that would preclude a precise and reproducible fit of the customized surgical guide. Furthermore, specimens were recorded as drop-outs during the experimental workflow due to: (1) procedural complications, such as bone fractures during implant placement or instability of the surgical guide (*n* = 4), and (2) technical data integrity issues, including loss of digital files during optical scanning, CBCT, or US image acquisition (*n* = 2). This resulted in a final sample of 18 hemimandibles for analysis.

### Standardized implant planning and surgical guide fabrication including the ultrasound probe holder

All samples were kept moist with damp towels in a cooler-box to prevent tissue desiccation. Immediately after collection, a calibrated intraoral scanner (Trios 5; 3Shape, Copenhagen, Denmark) was used to scan the dentition and mucosal contours. The resulting STL files were organized under a structured data-management plan. Subsequently, CBCT images were acquired using a high-resolution unit (GALILEOS; Dentsply Sirona, Charlotte, NC, USA) with a voxel size of 0.25 × 0.125 mm and parameters set to 98 kV and 25 mA, a scan time of 14 s, and a field of view (FOV) of 15 × 15 cm, following routine calibration procedures. Each hemimandible was positioned upright in a custom holder made of building blocks (LEGO, Billund, Denmark) and carefully aligned so that the occlusal plane was parallel to the CBCT device’s laser guide, ensuring consistent orientation across scans. The resulting images were stored as DICOM files. After the imaging procedures were completed, the mandibles were stored in a freezer within 8 hours of collection.

Implant planning and placement followed a standardized protocol. Optical and CBCT scans of each hemimandible were imported into dedicated implant treatment planning software (CoDiagnostiX v10.8; Dental Wings Inc., Montreal, Canada) and superimposed by matching hard-tissue landmarks via built-in registration algorithms; manual refinement was performed if the resulting superimposition did not meet the software-defined registration quality criteria or showed visual discrepancies. For each hemimandible, a single implant (Bone Level Implant Ø 3.3 × 10 mm NC; Straumann AG, Basel, Switzerland) was virtually positioned between the first premolar and canine, according to bone availability. The virtual planning protocol prioritized maximal bone engagement, maintained a minimum distance of 1.5 mm from adjacent teeth, and aimed to position the implant shoulder at the crestal bone level. Exposed implant threads were intentionally accepted in selected cases when complete intraosseous positioning was not feasible due to anatomical constraints, such as developing tooth germs and limited crestal bone volume, simulating clinical conditions associated with peri-implant bone loss.

**Fig. 1:**
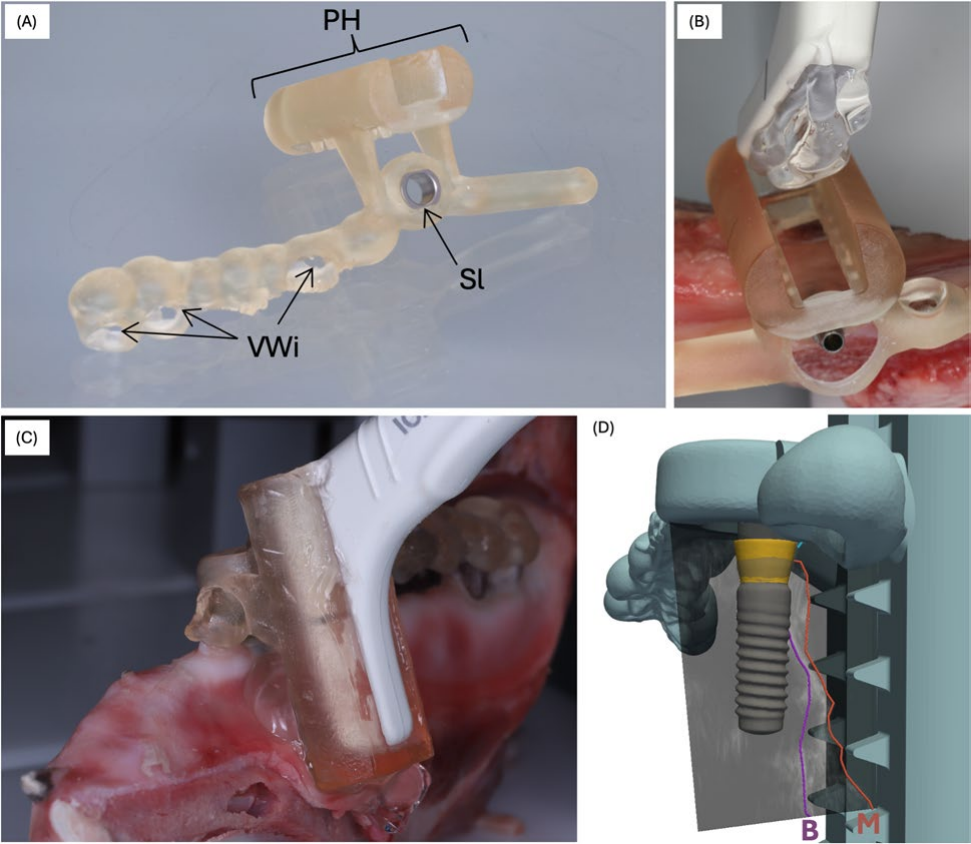
Ultrasound image acquisition for the test dataset. **A** Surgical guide with the customized ultrasound (US) probe holder (German patent application, DE 10 2024 113 730 A1) (PH, probe holder; Sl, stainless-steel drill sleeve; VWi, verification windows). **B** Modified surgical guide with the drill sleeve removed and an enlarged opening to accommodate the crown (crown not shown). **C** US test data acquisition on the lingual side of the mandible with the US probe in place. **D** Superimposition of the US image, expert annotation (B, Bone; M, Mucosa), and the surgical guide with adapted US probe holder

A tooth-supported surgical guide was then digitally designed for each hemimandible individually using the same software. Verification windows were incorporated to ensure accurate intraoperative seating. A newly developed US probe holder was integrated into the surgical guide using computer-aided design (CAD) and individually aligned sagittally and orthoradially parallel to the implant axis on the lingual side of the guide, thereby avoiding the muscle attachments present on the buccal aspect of the hemimandible (Fig. 1). The probe holder allows for targeted and reproducible positioning of the ultrasound probe in subsequent steps of the workflow.

Surgical guides were prepared for stereolithography (SLA) printing using Surgical Guide Resin (Formlabs Inc., Somerville, MA, USA) in proprietary software (PreForm; Formlabs Inc., Somerville, MA, USA) and then fabricated using an SLA-based 3D printer (Form 2; Formlabs Inc., Somerville, MA, USA). Post-processing involved cleaning in isopropanol, followed by 30 min of light curing at 60 °C. Stainless-steel drill sleeves (T-sleeve; Straumann AG, Basel, Switzerland) were inserted afterward.

### Surgical implant placement

The porcine mandibles were thawed for three hours prior to implant placement. A horizontal crestal incision was made with a No. 15 blade, followed by periosteum reflection and bone levelling with a periodontal chisel. With the surgical guide in place, implant beds were prepared according to the protocols defined by the implant treatment planning software, beginning with a water-cooled guided pilot drill (Ø 2.2 mm, 800 rpm) and concluding with a final drill (Ø 2.8 mm, 600 rpm), each used with its corresponding drill handle (Straumann AG, Basel, Switzerland).

Implants were inserted at 15 rpm and a torque of 35 N·cm with the surgical guide in place for orientation. Immediately after implant placement, a post-implantation optical scan was obtained with a scanbody attached (Scanbody Ø 3.3 mm NC; Straumann AG, Basel, Switzerland).

### Ultrasound image acquisition and dataset generation

Two calibrated operators acquired all B-mode US images using a 128-element, 7–18 MHz linear-array transducer (IO7–18; Alpinion Medical Systems, Seoul, South Korea) connected to a commercially available ultrasound system (X-CUBE 90; Alpinion Medical Systems, Seoul, South Korea). The operators were dentists and had completed a four-month pilot phase involving scans of 22 porcine hemimandibles to gain proficiency in US imaging techniques and instrument handling. Ultrasound gel (Alpinion Medical Systems, Seoul, South Korea) was applied to both the probe and the holder window before each scan to prevent air artifacts. During each scan, the frequency was held constant at 18 MHz, while depth, focus position, and gain were individually adjusted to optimize visualization of soft-tissue and bone interfaces. Images were acquired at a resolution of 1000 × 590 pixels at 8-bit grayscale and exported as TIFF files.

To facilitate both model training and testing, the ultrasound examination was divided into two distinct subsets: the training dataset and the test dataset. The test dataset was acquired using the guide-adapted US probe holder positioned at the implant site (Fig. 1). To simulate clinical conditions, a monolithic zirconia crown (IPS e.max ZirCAD Prime; Ivoclar, Schaan, Liechtenstein) was mounted on an abutment (Variobase; Straumann AG, Basel, Switzerland) and left in place throughout imaging. The surgical guide was modified by removing its drill sleeve and enlarging the opening to accommodate the crown (Fig. 1). This allowed the guide to seat seamlessly on the teeth while the crown remained on the abutment. Multiple images were acquired at the same position using varying depth, focus, and gain settings as described above to ensure optimal image quality. Following acquisition, a single representative US image was manually selected for each hemimandible (*n* = 18) from the dataset by two calibrated operators in consensus. Selection required the clear and distinct visualization of all six classes (mucosa, bone, implant, abutment, crown, and surgical guide). Consequently, the remaining 141 images were excluded from the test dataset.

For the training dataset, the tooth-supported components of the surgical guide were removed, retaining only the US probe holder. The holder was positioned freehand on the periodontium of adjacent teeth and the buccal site of the implant, oriented to capture as many of the six classes as possible. Multiple images were acquired at different positions on each hemimandible, resulting in a total of 606 images. This deliberate variability in probe placement generated a large and diverse training dataset.

### AI-based Ultrasound image segmentation

**Fig. 2:**
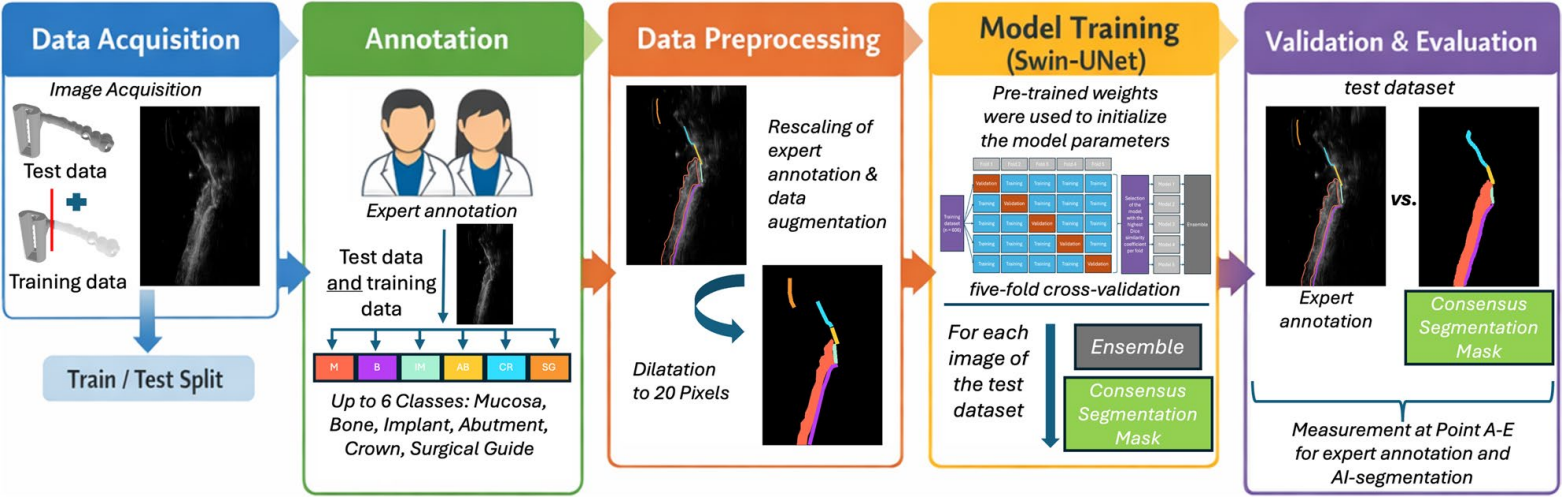
Schematic overview of the artificial intelligence-based ultrasound image segmentation workflow. Ultrasound (US) images were acquired and divided into a training dataset and a test dataset. On each image, up to six classes were annotated by experts: M, Mucosa; B, Bone; IM, Implant; AB, Abutment; CR, Crown; SG, Surgical guide. During preprocessing, annotations were dilated and data augmentation was applied. The Swin‑UNet model was initialized with pre‑trained weights and trained using five‑fold cross‑validation, resulting in an ensemble of five models. This ensemble was subsequently applied to the test dataset to produce consensus segmentation masks, from which mucosal thickness measurements were calculated and quantitatively compared with the expert annotations

The workflow for the AI-based image segmentation is illustrated in Fig. 2 and described in detail in the following sections. The descriptions follow the CLAIM Guidelines for reporting of artificial intelligence in medical imaging studies.

### Reference standard, expert annotation and model architecture

The reference standard for AI-based segmentation was established through expert annotation, performed independently by two trained experts, each with four months of experience in dental sonography. To mitigate inter-rater variability, annotators followed a standardized protocol for identifying and labelling up to six distinct classes: mucosa, bone, implant, abutment, crown, and surgical guide. Image annotation was carried out on a 22” LED monitor (Fujitsu TFT B22-8 WE Neo; Fujitsu, Tokyo, Japan) at the Department of Prosthodontics using a locally hosted instance of the Computer Vision Annotation Tool (CVAT v2.2; open source, originally developed by Intel, Santa Clara, CA, USA), a platform commonly used for creating labeled datasets. A deep learning approach based on the Swin-UNet architecture [48] was utilized for semantic segmentation of the US images. The model was configured with a patch size of 4 and a window size of 12. During preprocessing, images were rescaled from their original resolution of 1000 × 590 pixels to 384 × 384 pixels, and random brightness variations were applied for data augmentation.

### Data partition and model training

Before model training, the polyline annotations were dilated to a width of 20 pixels to permit the use of an overlap-based loss function, implemented as an equally weighted combination of Dice loss and cross-entropy loss.

**Fig. 3:**
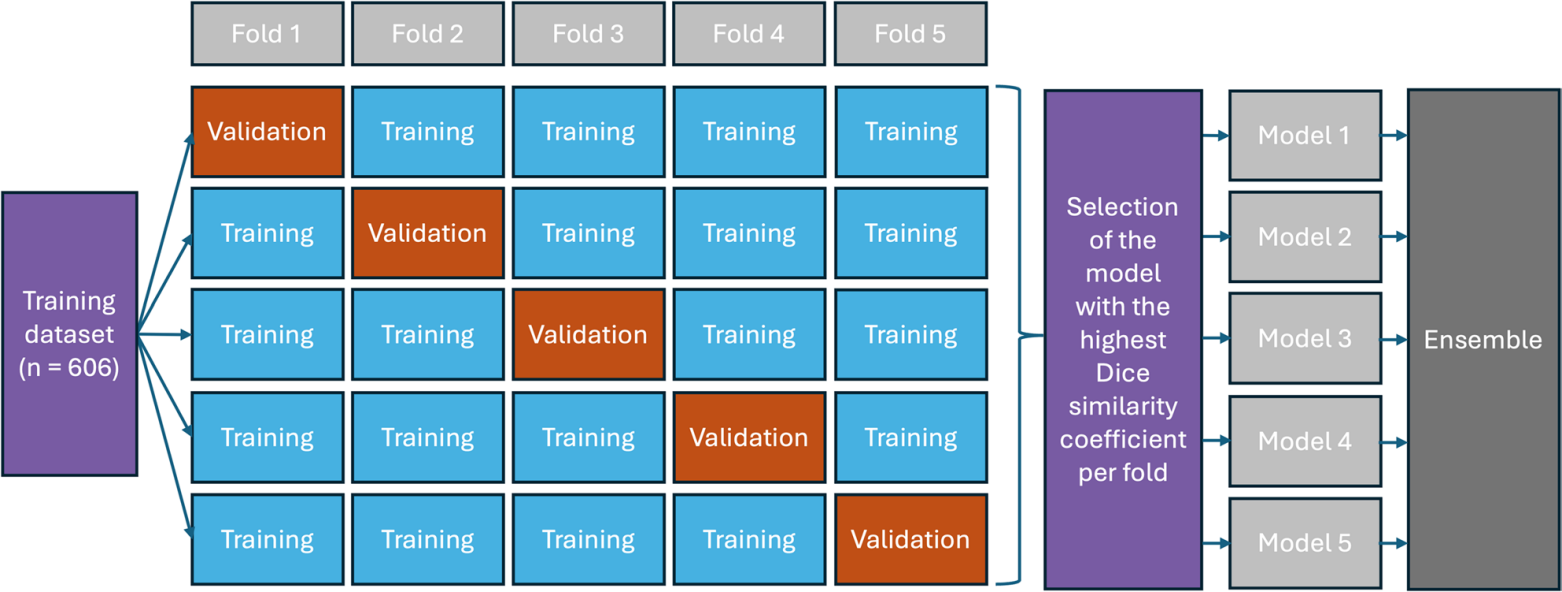
Five-fold cross-validation scheme

Training was performed on a dedicated graphics processing unit (GPU) cluster (NVIDIA H100 GPU; NVIDIA Corporation, Santa Clara, CA, USA), using a five-fold cross-validation scheme (Fig. 3). Given the moderate dataset size, five folds were considered a reasonable compromise between reliable performance estimation and computational efficiency. Alternative approaches, such as leave-one-out cross-validation or using fewer folds, would either increase computational cost or reduce the stability of performance estimates.

**Fig. 4:**
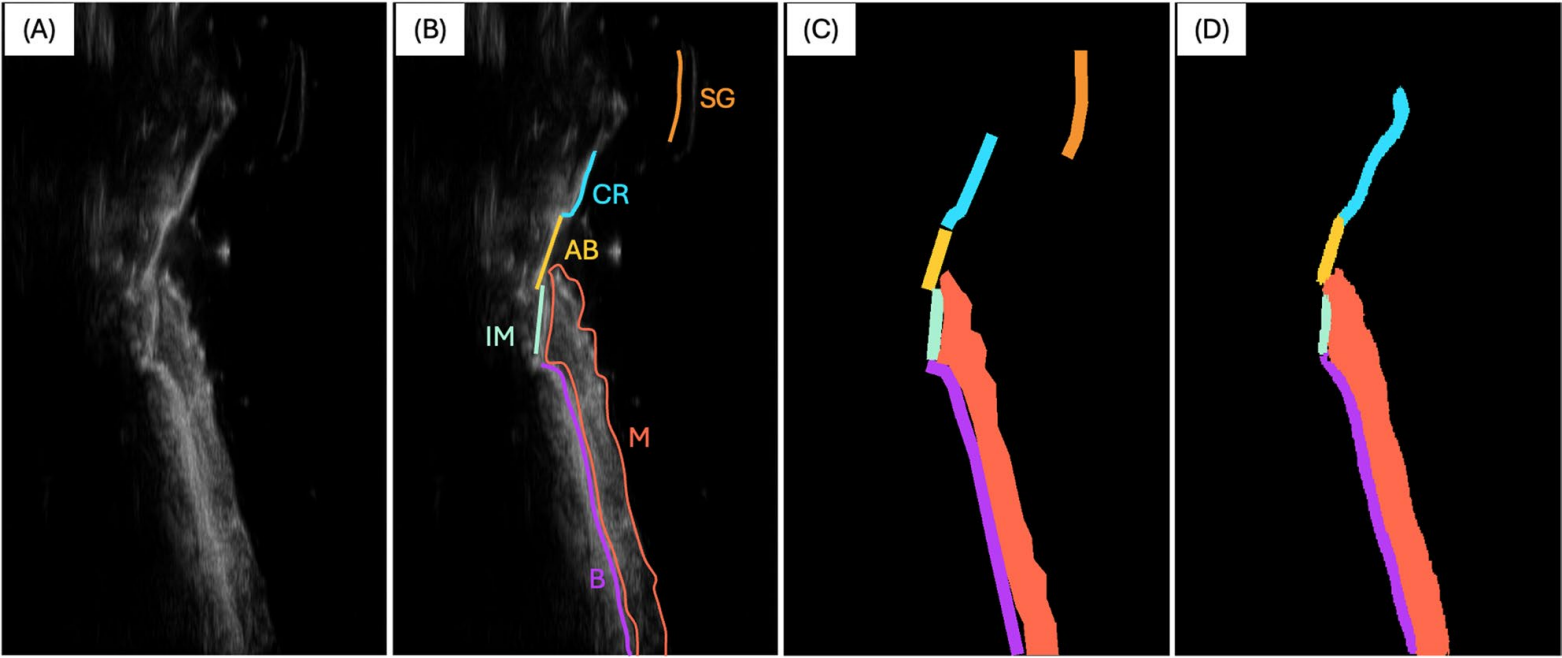
Processing of the ultrasound test dataset. **A** Ultrasound (US) image (test data). **B** Expert‑annotated US image (M, Mucosa; B, Bone; IM, Implant; AB, Abutment; CR, Crown; SG, Surgical guide). **C** Dilated expert‑annotated US image. **D** Prediction of the final ensemble (consensus segmentation mask)

The training partition was randomly split into five equal subsets; in each fold, four subsets were used for training while the remaining subset served for model tuning, allowing each image to contribute to both model training and tuning. The Swin-UNet model was then trained with a learning rate of 0.0003, a batch size of 32, and 50 epochs per fold. Parameters were initialized using pre-trained weights from a large-scale image classification dataset (ImageNet; Stanford Vision Lab, Stanford, CA, USA). After each fold, the model achieving the highest Dice similarity coefficient (DSC) on the validation set was selected, ensuring robust and generalizable segmentation performance. Upon completion of cross-validation, the five resulting models were combined into an ensemble. Final segmentation was performed by averaging the pixel-wise predictions from all five models, producing a separate consensus segmentation mask for each image of the test dataset (Fig. 4). This ensemble approach mitigates individual model variance and typically yields more accurate and stable segmentations.

### Spatial registration and mucosal thickness measurement

The spatial relationship between the US image and the implant was conceptually established via the US probe holder integrated into the surgical guide. As minor deviations between the planned and the actual implant position are inevitable even in guided implant surgery, the spatial relationship had to be recalculated:

1 $$\:\begin{array}{c}{{}_{\:}{}^{US\:Image}T}_{Implant}={{}_{\:}{}^{US\:Image}T}_{Guide}\:\cdot\:{{}_{\:}{}^{Guide}T}_{Implant}\end{array}$$

The implant position in the US coordinate system was determined using geometric information from both the surgical guide and the surrounding bone surfaces (Eq. 1). To achieve this, the post-implantation optical scan with a scanbody attached was matched to a reference scan of the implant with the same scanbody attached, acquired outside the hemimandible. Registration of the two datasets based on the scanbody geometry yielded the implant position relative to the bone.

Subsequently, the post-implantation optical scan was registered to the STL model of the surgical guide using surface geometry. This registration established the spatial relationship between the guide and the bone, thereby determining the position of the guide relative to the implant ($\:{{}_{\:}{}^{Guide}T}_{Implant})$. This transformation was further refined by orienting the surgical guide within the US image through alignment with visible guide structures.

**Fig. 5:**
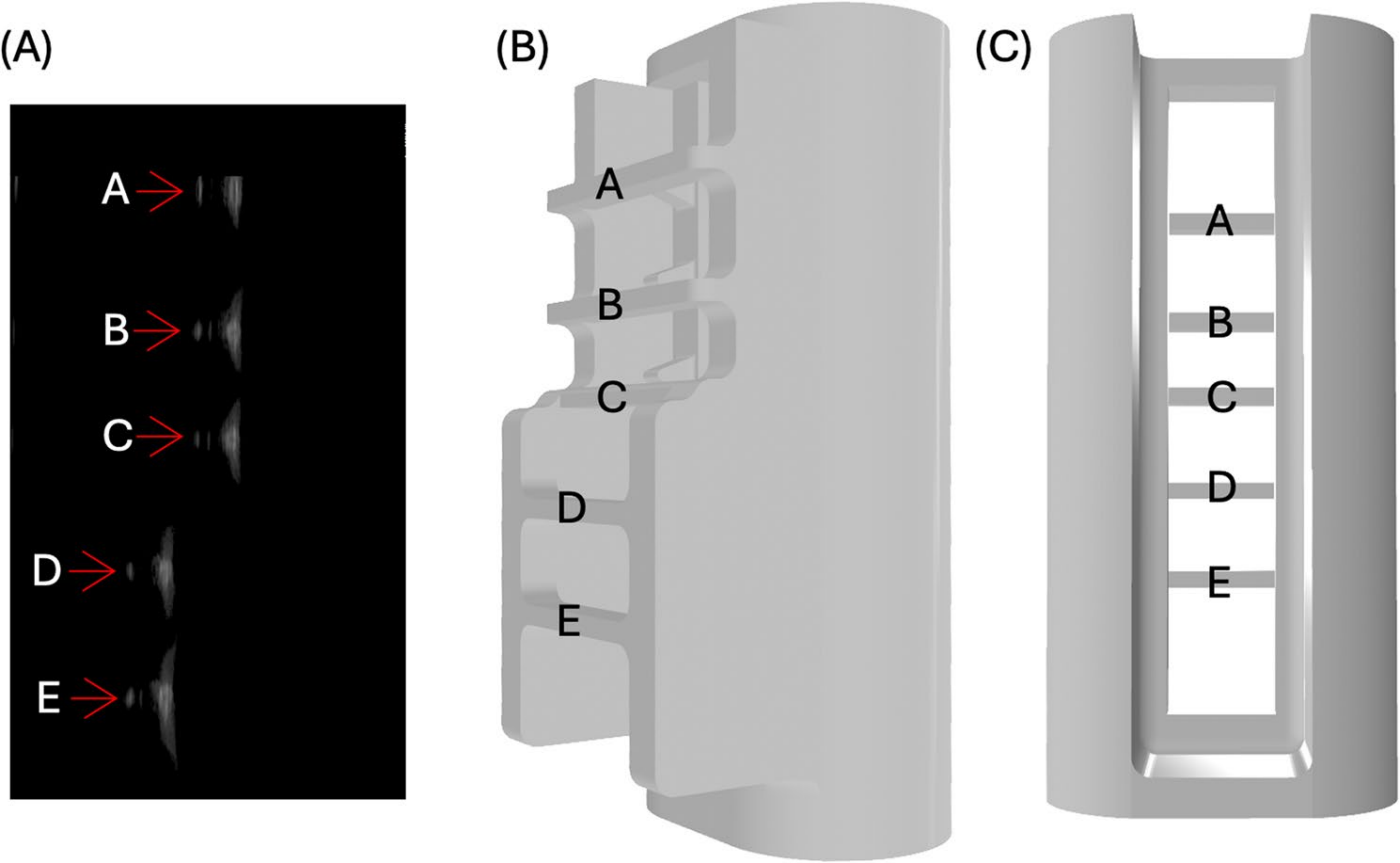
Calibration body with corresponding ultrasound image. **A** Ultrasound (US) image acquired with the US probe positioned in the calibration body immersed in a water bath. The US markers A–E are visible in the image. **B** Side view of the calibration body showing the US markers A–E aligned vertically at the heights of measurement points A–E. **C** Front view of the calibration body

A calibration body (Fig. 5) was then used to map the positions of the five anatomically standardized measurement points (A–E) in the US images, to derive a calibration matrix for converting pixel distances to metric units (mm), and to determine the position of the guide in the US Image $\:{({}_{\:}{}^{US\:Image}T}_{Guide})$. This allowed the implant position to be identified within the US image ($\:{{}_{\:}{}^{US\:Image}T}_{Implant})$. For each image in the test dataset, MT values were computed at points A–E from both the consensus segmentation masks generated by the AI model and the expert-annotated US images, allowing direct comparison between AI-segmented and expert-annotated MT measurements. At each measurement point, MT was defined as the distance along the image axis from the mucosal surface to the underlying bone or implant interface, yielding quantitative values that can be reproducibly obtained without manual annotation.

### Mucosal thickness measurement via transgingival probing

**Fig. 6:**
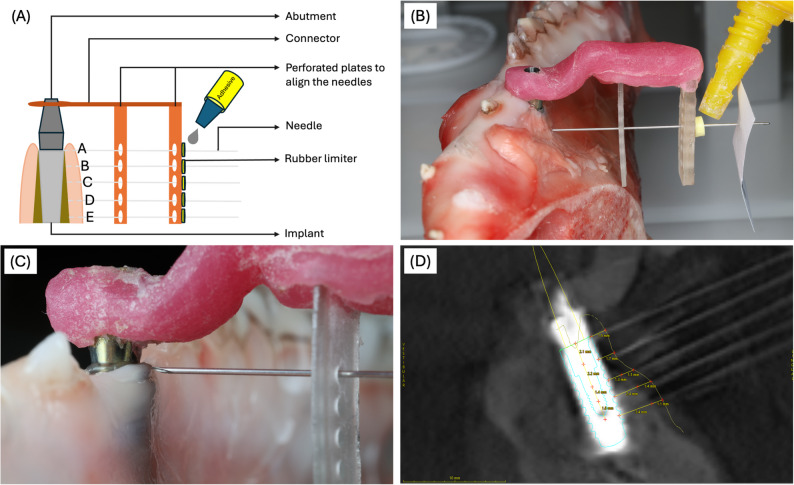
Mucosal thickness measurement using transgingival probing and cone*-*beam computed tomography. **A** Measurement device for transgingival probing (TP) and cone‑beam computed tomography (CBCT) with reproducible horizontal and vertical alignment at standardized points (A–E). **B** Fixation of the rubber limiter. **C** Needle in contact with the mucosa at point A. **D** Superimposition of optical scan and CBCT with mucosal thickness measurements in coronal slices at points A–E

A single operator performed TP for MT measurement. Needles (G25 × 1.5”, Ø 0.5 × 40 mm) were inserted through perforated plates attached to the implant to ensure reproducible alignment at standardized measurement points (A–E). These points were aligned vertically at predetermined intervals from the crestal edge in an apical direction (Fig. 6). Under 3× magnification, the needles were advanced until mucosal contact, and a rubber limiter was attached and fixed with cyanoacrylate adhesive. Using an optical measuring projector (IM-8000; Keyence Corporation, Osaka, Japan) with an accuracy of ± 0.01 mm, the distance from the needle tip to the limiter was recorded. The procedure was repeated for bone or implant contact, and MT was calculated as the difference between the two measurements.

### Mucosal thickness measurement via CBCT

After TP, a CBCT scan was acquired using the same parameters as in the pre-implant placement scan, with the needles in bone contact (Fig. 6). The needles served as fixed physical trajectories for the measurements, ensuring that MT measurements were performed at the exact spatial locations used for TP and US. The CBCT scans and the corresponding post-implantation optical scans were superimposed in the implant treatment planning software (CoDiagnostiX). All measurements were performed directly within this software by a single calibrated operator who had completed a certified multi-month CBCT training course.

A standardized protocol was applied: coronal slices were selected in which the implant appeared at full diameter and the measurement needles were clearly visible. If implant-related artifacts obscured the bone surface in a given slice, the adjacent slice with clearly visible bone and needle was used. If artifacts or insufficient image quality precluded a reliable evaluation, the CBCT scan was repeated to ensure consistent data quality. Measurement lines were drawn along the trajectories of the needles and extended to the mucosal contour visualized in the optical scan (Fig. 6). In sites with complete intraosseous implant positioning, measurements were taken from the bone surface to the mucosa; in sites with exposed coronal implant threads, measurements were taken from the implant surface to the mucosa. MT was measured at the five standardized points (A–E) according to this protocol.

### Statistical analysis

All analyses were conducted using a statistical software package (SPSS Statistics v29.0.2.0; IBM Corp., Armonk, NY, USA). An a priori power calculation was performed using power analysis software (G*Power v3.1; HHU, Düsseldorf, Germany) based on a one-group repeated-measures ANOVA (within-subject factor “method”; *f* = 0.32; α = 0.05; 1 − β = 0.80; *r* = 0.5) as an approximation to a linear mixed-effects model (LMM). The calculation was based on pilot data (*n* = 22 hemimandibles), suggesting a small-to-moderate effect of measurement method (estimated Cohen’s *f* ≈ 0.32), and indicated that 18 porcine hemimandibles were required.

An LMM was fitted to log-transformed data, with mandible as a random intercept to account for variability across specimens (level-2 clustering), and measurement method (TP, CBCT, expert-annotated US) and measurement point (A–E) as fixed effects. Measurement point was included solely to control for systematic location effects. The inclusion of a random slope for measurement method was evaluated using the Akaike information criterion (AIC) and retained if it improved model fit. Interactions between method and measurement point were explored but omitted if non-significant. Residual normality and homoscedasticity were assessed using Q-Q plots and scatterplots; log transformation improved residual normality and was therefore used in the LMM. Statistical significance was set at *p* < 0.05.

Agreement for predefined method pairs (expert-annotated US versus TP, expert-annotated US versus CBCT, expert-annotated US versus AI-segmented US) was assessed using Bland–Altman plots and intraclass correlation coefficients (ICC(2,1), two-way random-effects model, absolute-agreement; 95% confidence interval (CI)). Acceptable limits of agreement (LoA) were defined a priori as ± 0.5 mm, based on the practical resolution of TP in clinical use. Bland–Altman and ICC analyses were performed on the original data scale to preserve interpretability; histograms of paired differences were inspected to assess approximate normality.

## Results

### Sample characteristics

A total of 18 porcine hemimandibles were included in the final analysis, each contributing five standardized measurement points (A–E) assessed by TP, CBCT, and US, yielding 90 observations per method. For the AI-segmented US group, eight measurements were excluded (six at point A, one at point B, and one at point C), because the AI model produced implausible 0 mm values in these instances. Consequently, the final dataset comprised 352 valid observations (90 TP, 90 CBCT, 90 expert-annotated US, and 82 AI-segmented US) for statistical evaluation.

### Evaluation of mucosal thickness measurement methods using a linear mixed model (LMM)

Including a random slope for measurement method improved the model (ΔAIC = 38.86). The final model therefore included both a random intercept and a random slope at the mandible level. The method versus point interaction was non-significant (*p* = 0.627) and was omitted. Q-Q plots from the log-transformed data suggested approximate normality (see Additional file 2), and scatterplots showed homoscedasticity (see Additional file 3).

The fixed effect of measurement method was not statistically significant (*p* = 0.105), indicating no global difference between TP, CBCT, and expert-annotated US (Table 1). Pairwise comparisons revealed that CBCT yielded higher MT values compared with TP (log-mean difference 0.148; *p* = 0.035), while expert-annotated US measurements were not significantly different from TP (log-mean difference 0.067; *p* = 0.328). These pairwise findings are exploratory and should be interpreted cautiously given the non-significant omnibus test.

**Table 1:** Results of the linear mixed-effects model: fixed and random effects, and model fit indices

Fixed Effects	Estimate	*SE*	95% CI		*p*-value	*df*
			Lower Bound	Upper Bound		
Measurement method (TP, CBCT, expert-annotated US)	—	—	—	—	0.105	2.36
[TP (Ref.)] Intercept^a^	0.722	0.062	0.600	0.845	< 0.001	90.46
CBCT–TP	0.148	0.068	0.011	0.285	0.035	36
expert-annotated US–TP	0.067	0.068	−0.070	0.204	0.328	36

Random Effects	Variance	*SE*	95% CI			
			Lower Bound	Upper Bound		
Mandible (Intercept)^b^	0.009	0.008	0.002	0.054		
Mandible (Slope)^b^	0.028	0.028	0.014	0.055		
Residual	0.068	0.068	0.056	0.082		

Model Fit Index						Value
Akaike information criterion (AIC)						129.32
Bayesian information criterion (BIC)						165.31
ΔAIC (with versus without random slope)						38.86

The log-transformed mucosal thickness served as the dependent variable

*Abbreviations*: *TP* transgingival probing, *CBCT* cone-beam computed tomography, *US* ultrasound, *LMM* linear mixed-effects model, *CI* confidence interval, *AIC* Akaike information criterion, *BIC* Bayesian information criterion, *SE* standard error, *df* degrees of freedom

^a^TP served in the LMM as the statistical comparator. All corresponding estimates indicate the mean deviation from TP

^b^A random intercept and a random slope for the factor “method” were included at the mandible level

### Bland–Altman analysis of method agreement

Histograms of paired differences and Q-Q plots approximated a normal distribution (see Additional file 4). Visual inspection of Bland–Altman plots showed no evidence of proportional bias and an approximately uniform scatter across the measurement range, suggesting homoscedasticity.

**Fig. 7:**
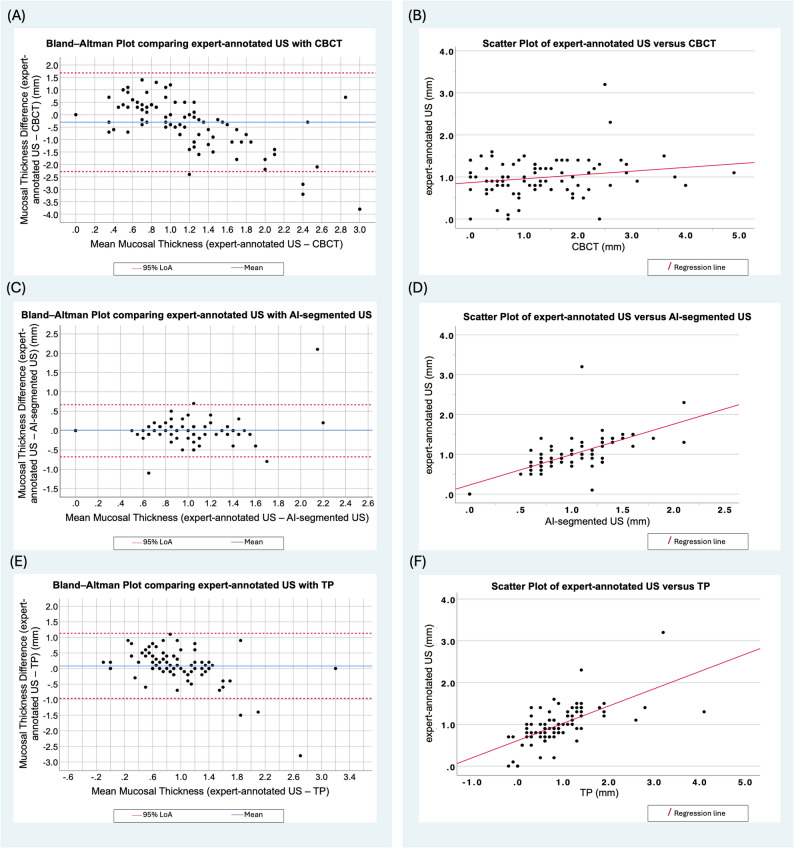
Bland–Altman plots and scatterplots. **A** Bland–Altman plot comparing expert‑annotated Ultrasound (US) with cone‑beam computed tomography (CBCT). **B** Expert‑annotated US versus CBCT. **C** Bland–Altman plot comparing expert‑annotated US with artificial intelligence (AI) segmented US. **D** Expert‑annotated US versus AI‑segmented US. **E** Bland–Altman plot comparing expert‑annotated US with transgingival probing (TP). **F** Expert‑annotated US versus TP

In all three comparisons, the LoA extended beyond the predefined ± 0.5 mm tolerance window (Fig. 7), indicating that individual paired differences occasionally exceeded this threshold. For expert-annotated US − TP, the mean difference was 0.08 mm; however, 18% of paired differences exceeded ± 0.5 mm (95% LoA: −0.96 mm to + 1.13 mm). Expert-annotated US − CBCT showed a larger mean difference of − 0.30 mm with wider LoA (− 2.29 mm to + 1.68 mm). In contrast, expert-annotated US − AI-segmented US deviated by only − 0.01 mm on average (95% LoA: −0.68 mm to + 0.67 mm).

### Reliability of mucosal thickness measurements assessed by intraclass correlation coefficients (ICC)

The normality of paired differences was assessed by Q-Q plots. Using the classification proposed by Koo & Li [49] (ICC < 0.50 = poor, 0.50 to 0.75 = moderate, 0.75 to 0.90 = good, and > 0.90 = excellent) agreement was moderate for expert-annotated US versus TP and for expert-annotated US versus AI-segmented US, and poor for expert-annotated US versus CBCT (Table 2).

**Table 2:** Intraclass correlation coefficients comparing expert-annotated ultrasound with transgingival probing, cone-beam computed tomography, and AI-segmented ultrasound

*n*	Comparison	ICC(2,1)	95% CI	
			Lower Bound	Upper Bound
90	Expert-annotated US versus TP	0.579	0.424	0.701
90	Expert-annotated US versus CBCT	0.141	−0.053	0.329
82	Expert-annotated US versus AI-segmented US	0.669	0.529	0.773

*Abbreviations*: *n* number of paired measurements, *US* ultrasound, *TP* transgingival probing, *AI* artificial intelligence, *CBCT* cone-beam computed tomography, *ICC* intraclass correlation coefficient, *CI* confidence interval

While the LMM did not show a statistically significant difference between expert-annotated US and TP on average, the moderate ICC values indicate inherent variability in single measurements. Similarly, the ICC between expert-annotated and AI-segmented US measurements indicates that moderate variability is also present between these two methods.

## Discussion

Our hypothesis was partially supported. In this controlled ex vivo model, agreement differed across comparators, with US aligning more closely with TP than with CBCT; however, the data do not support claims of superiority of US over either comparator for peri-implant MT assessment.

The main contribution of this study is a standardized workflow for reproducible, non-invasive peri-implant US assessment after implant placement, combining a customized probe holder to control transducer positioning and coupling with an AI model for automated image analysis. This approach aims to simplify acquisition and interpretation to support broader usability in dentistry, where sonographic image interpretation is not routinely part of dental training [45].

AI-based US segmentation produced MT measurements close to expert annotation on average, consistent with prior machine-learning applications in periodontal soft-tissue imaging [46]. However, only moderate agreement at the individual measurement level and the occurrence of implausible outputs requiring exclusion suggest that the current model is not yet suitable for unsupervised use and still requires expert oversight, particularly in low-contrast regions with sharply tapering soft-tissue contours. For clinical translation, automated quality control to flag likely segmentation failures may be essential.

Although dental-specific US probes have been described [50, 51], compact probes suitable for routine intraoral use remain uncommon. Therefore, a hockey-stick transducer adapted from arthroscopic applications was used as a pragmatic option in the absence of dedicated intraoral probes. Because small deviations in angulation and coupling can change the spatial relationship between the transducer, surgical guide, bone surface, and implant, standardized acquisition is critical for reliable MT assessment. The customized probe holder reduced variability related to probe positioning and coupling—factors that are often not standardized or reported in previous work [28, 52]—and may be particularly valuable for longitudinal follow-up, where within-site changes are evaluated under comparable conditions [8]. At the same time, the workflow proved technically sensitive: minor deviations from the planned implant position necessitated two registration steps (bone-to-implant and bone-to-guide) to ensure spatial correspondence between modalities. This highlights the importance of further improving positioning robustness. Increasing the number of supporting teeth to enlarge the contact area could improve stability of the guide–probe holder assembly and reduce angular deviations [53]; in vivo, this may be advantageous compared with the predominantly linear support available in the porcine hemimandible model. Additionally, designing the probe holder based on postoperative scans could reduce the need for registration and further improve measurement consistency.

Future work should focus on in vivo validation of the probe holder, improvements in positioning precision (e.g., sensor-based monitoring or integrated correction algorithms), and development of more robust AI-based segmentation using larger, diverse clinical datasets. Smaller, dental-specific intraoral US transducers are needed to facilitate clinical application across different anatomical regions. Complementary US modalities such as power Doppler may be explored to support longitudinal peri-implant monitoring and assessment of inflammatory changes; in addition, standardized acquisition protocols and training requirements for clinical personnel should be defined to support implementation of US in implant dentistry.

Limitations should be considered when interpreting the findings of this study. First, it was based on an experimental porcine model under controlled conditions with a small sample size, limiting extrapolation to clinical imaging. Second, intraoral confounders (e.g., saliva, patient motion, and restricted probe access—particularly in posterior regions) were not present and may attenuate the US signal and compromise image quality in vivo [52]. Third, the custom probe holder remains an experimental prototype used under ex vivo conditions and has not yet been clinically validated; it is currently optimized for anterior and buccal sites, and the findings do not generalize to freehand intraoral US probe placement. Fourth, the AI model was developed on a limited training dataset and requires external validation on larger, more diverse in vivo cohorts. Fifth, manual selection of a representative US image per site for the test dataset may have introduced operator-dependent variability despite consensus selection by calibrated operators. Finally, in the absence of a true reference standard, agreement between methods must be interpreted in light of comparator limitations: TP has limited practical resolution and is susceptible to technique-related variability [24], while CBCT-derived MT measurements around implants are susceptible to metal-induced artifacts and limited soft-tissue contrast [35].

## Conclusions

Under controlled ex vivo conditions, US standardized with a custom probe holder showed moderate agreement with TP for peri-implant MT assessment. AI-based segmentation approximated expert annotation, whereas CBCT showed limited agreement with US. These findings do not imply equivalence or clinical interchangeability of the methods. This integrated workflow remains a proof-of-concept and requires further in vivo validation.

## Supplementary Information

Additional file 1: Patent Application of the Ultrasound Probe Holder.

Additional file 2: LMM Q-Q-Plot of Residuals Log-Data and Raw-Data.

Additional file 3: LMM Scatterplots Homoscedasticity Log-Data.

Additional file 4: Histograms of Residuals Q-Q-Plots.

## Data Availability

The data that support the findings of this study are available from the corresponding author upon reasonable request.

## Abbreviations

MT: Mucosal Thickness
US: Ultrasound
TP: Transgingival Probing
CBCT: Cone-beam Computed Tomography
AI: Artificial Intelligence
ICC: Intraclass Correlation Coefficient
CI: Confidence Interval
STL: Standard Tessellation Language (3D model file format)
FOV: Field of View
DICOM: Digital Imaging and Communications in Medicine
CAD: Computer-Aided Design
SLA: Stereolithography (additive manufacturing / 3D printing technology)
PH: Probe Holder
VWi: Verification Windows
Sl: Stainless-steel Drill Sleeve
B-mode: Brightness Mode (ultrasound imaging)
TIFF: Tagged Image File Format
CLAIM: Checklist for Artificial Intelligence in Medical Imaging
SG: Surgical Guide
CR: Crown
AB: Abutment
IM: Implant
M: Mucosa
B: Bone
CVAT: Computer Vision Annotation Tool
DSC: Dice Similarity Coefficient
GPU: Graphics Processing Unit
ANOVA: Analysis of Variance
LMM: Linear Mixed-Effects Model
LoA: Limits of Agreement
AIC: Akaike Information Criterion
*SE*: Standard Error
*df*: Degrees of Freedom
BIC: Bayesian Information Criterion
