# Supplementary material for: Ultrasound-based assessment of peri-implant mucosal thickness: an ex vivo comparative study with artificial intelligence-assisted image analysis
Source: BMC Oral Health. 2026 Jun 27;26:1215. doi: 10.1186/s12903-026-08665-0 (PMC13343591; doi:10.1186/s12903-026-08665-0)
Supplement: Supplementary file 1 — Additional file 1: Patent Application of the Ultrasound Probe Holder. [file 12903_2026_8665_MOESM1_ESM.pdf]

(19) Deutsches Patent- und Markenamt

(10) **DE 10 2024 113 730 A1** 2025.11.20

(12) **Offenlegungsschrift**

(21) Aktenzeichen: **10 2024 113 730.4**
(22) Anmeldetag: **16.05.2024**
(43) Offenlegungstag: **20.11.2025**

(51) Int Cl.: ***A61C 19/04*** (2006.01)

(71) Anmelder:
Rheinisch-Westfälische Technische Hochschule Aachen, abgekürzt RWTH Aachen, Körperschaft des öffentlichen Rechts, 52062 Aachen, DE

(74) Vertreter:
Eisenführ Speiser Patentanwälte Rechtsanwälte PartGmbB, 28217 Bremen, DE

(72) Erfinder:
Großhausen, Juliana Marotti, Dr., 79539 Lörrach, DE; Radermacher, Klaus, Prof. Dr., 52222 Stolberg, DE; Fuente Klein, Matias de la, 52066 Aachen, DE; Brößner, Peter, 52070 Aachen, DE; Hohlmann, Benjamin, 52064 Aachen, DE

(56) Ermittelter Stand der Technik:

**DE**	**199 52 962**	**A1**
**US**	**2005 / 0 070 797**	**A1**
**US**	**2018 / 0 042 696**	**A1**
**US**	**2019 / 0 125 297**	**A1**
**EP**	**3 326 573**	**A1**

NGUYEN, K. C. T. [u.a.]: Alveolar bone segmentation in intraoral ultrasonographs with machine learning. In: Journal of Dental Research, Vol. 99, 2020, No. 9, S. 1054-1061. - ISSN 0022-0345 (P); 1544-0591 (E). DOI: 10.1177/0022034520920593. URL: https://journals.sagepub.com/doi/reader/10.1177/0022034520920593 [abgerufen am 2024-07-22]

Rechercheantrag gemäß § 43 PatG ist gestellt.

Die folgenden Angaben sind den vom Anmelder eingereichten Unterlagen entnommen.

(54) Bezeichnung: **Vorrichtung für eine intraorale Positionierung eines Ultraschallkopfes**

(57) Zusammenfassung: Die Erfindung betrifft eine Vorrichtung (1) für eine reproduzierbare intraorale Positionierung eines Ultraschall-kopfes (2), mit einer Dentalschiene (4), die einen Dentalschienenkörper (6) aufweist, der an einer intraoralen, anatomischen Struktur (8) temporär positionierbar ist und dessen intraorale Positionierung durch die intraorale, anatomische Struktur (8) definiert ist, wobei der Dentalschienenkörper (6) die intraorale, anatomische Struktur (8) derart nachbildet, dass der Dentalschienenkörper (6) in exakt einer räumlich definierten Position formschlüssig an die intraorale, anatomische Struktur (8) positionierbar ist. Die Dentalschiene (4) weist einen Aufnahmeabschnitt (10) zum Aufnehmen des Ultraschallkopfes (2) auf, wobei der Aufnahmeabschnitt (10) einen Schalldurchtrittsbereich (12) aufweist, durch den Ultraschallwellen von dem Ultraschallkopf (2) zu einem darzustellenden Abschnitt (14) der intraoralen, anatomischen Struktur (8) hindurchtreten können. Der Aufnahmeabschnitt (10) weist wenigstens ein Positionierelement (16) aufs, mit dem der Ultraschallkopf (2) in Kontakt gebracht werden kann, um die Pose des Ultraschallkopfes (2) relativ zur Dentalschiene (4) zu definieren.

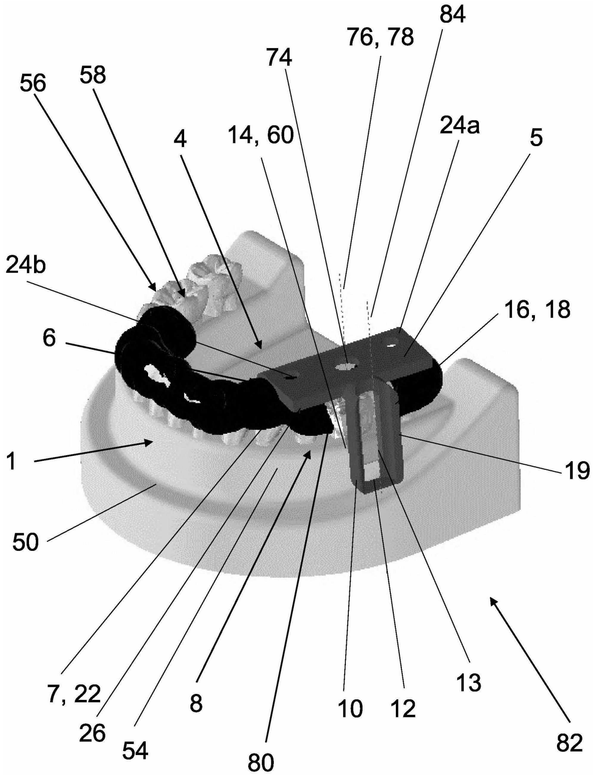

Beschreibung

[0001] Die Erfindung betrifft eine Vorrichtung für eine intraorale Positionierung eines Ultraschallkopfes, ein Verfahren zum Herstellen einer Vorrichtung für die intraorale Positionierung eines Ultraschallkopfes und eine Verwendung einer Vorrichtung für eine intraorale Positionierung eines Ultraschallkopfes zum Aufnehmen von intraoralen Ultraschallbilden eines darzustellenden Abschnitts einer intraoralen, anatomischen Struktur mit einem Ultraschallkopf.

[0002] Der Verlust eines Zahns oder mehrerer Zähne kann bei Patienten durch ein Zahnimplantat bzw. Zahnimplantate behandelt werden. Zahnimplantate werden eingesetzt, um die Zahnfunktion, die Ästhetik und/oder die Aussprache von Patienten wiederherzustellen. Zahnimplantate werden an den betreffenden Stellen in den Kieferknochen verankert und ersetzen damit vollständig die Zahnwurzel.

[0003] Ein erfolgreicher Einsatz eines Implantats hängt von Faktoren wie z. B. dem Vorhandensein von Knochen, der Knochenqualität, dem Zahnfleisch und/oder der Mundhygiene ab. Komplikationen bei Patienten mit Zahnimplantaten können sich insbesondere dann ergeben, wenn Infektionen im Implantationsbereich auftreten, sog. Periimplantitis. Periimplantitis ist ein entzündlicher Prozess, der in den meisten Fällen asymptomatisch ist, das Gewebe um ein Implantat angreift und mit Vereiterung, erhöhter Sondierungstiefe und Knochenresorption einhergeht und zum Verlust des Implantats führen kann.

[0004] Zum Behandeln von Periimplantitis werden chirurgische und nicht-chirurgische Maßnahmen ergriffen, wobei die ergriffenen Maßnahmen meist von dem Schweregrad der Entzündung abhängig sind. Nicht-chirurgische Behandlungsansätze führen jedoch oft nicht zu den gewünschten Ergebnissen und bei chirurgischen Behandlungsansätzen besteht die Gefahr von Folgekomplikation, bspw. ästhetische Beeinträchtigungen als Folge von Zahnfleisch- bzw. Weichgeweberückgang. Insofern ist insbesondere die Vorsorge von besonderer Bedeutung, um die frühen Anzeichen einer periimplantären Gewebeentzündung (Peri-Mukositis) zu erkennen und damit, die Periimplantitis zu verhindern. Um frühzeitig Veränderungen im Implantationsbereich, insbesondere Veränderungen des Knochens und/oder des Weichgewebes, zu erkennen, ist eine engmaschige Überwachung des Patienten notwendig.

[0005] Zum Beurteilen bzw. Messen des Knochens werden üblicherweise bildgebende Verfahren mit Röntgenstrahlung wie die periapikale Röntgenaufnahme eingesetzt. Problematisch an Röntgenstrahlung ist jedoch, dass diese ionisierend ist und insofern Veränderungen am menschlichen Organismus hervorrufen kann. Die periapikale Röntgenaufnahme hat eine niedrige Strahlendosis, liefert aber nur ein 2D-Bild und ermöglicht die Beurteilung des Knochenniveaus nur auf der mesialen und distalen Seite des Implantats, ohne Informationen über den bukkalen Knochen zu liefern, der in der Regel am dünnsten und am anfälligsten für erste Anzeichen von Knochenverlust ist. Die Digitale Volumentomographie (DVT) gilt als Goldstandard für die 3D-Bewertung des Knochens, da die Untersuchung eine hohe Auflösung und eine geringere Strahlendosis als die Computertomographie bietet. Allerdings gilt die Strahlendosis der DVT immer noch als hoch, so dass ihr Einsatz bei regelmäßigen Nachuntersuchungen vermieden werden muss. Außerdem treten bei der DVT in der Regel Metallartefakte auf, die zu einer Unterschätzung der Knochendicke führen können, was der Befund beeinträchtigt.

[0006] In der Medizin sind zudem bildgebende Verfahren mittels Ultraschall (US) bekannt. Bildgebende Verfahren mittels Ultraschall haben den Vorteil, dass sie nicht-invasiv sind und keine ionisierende Strahlung umfassen, so dass für wiederholte Kontrolluntersuchungen die Ultraschallbildgebung unbedenklich und vor diesem Hintergrund der Röntgenbildgebung vorzuziehen ist. Außerdem erlaubt die Ultraschallbildgebung Aufnahmen in Echtzeit, die kostengünstig und mit transportablen Geräten möglich sind.

[0007] Aus dem Stand der Technik sind Studien bekannt, die zeigen, dass Ultraschall auch bei der Vermessung intraoraler Knochen und/oder der Dicke des Weichgewebes eingesetzt werden kann.

[0008] In der Publikation Alveolar Bone Segmentation in Intraoral Ultrasonographs with Machine Learning - Nguyen KCT, et al., Journal of Dental Research, May 2020, doi: 10.1177/0022034520920593 ist beschrieben, dass Zähne von erwachsenen Patienten, die sich einer zahnärztlichen Behandlung unterzogen haben, mit einem transportablen Ultraschallscanner freihändig gescannt werden. Ein maßgefertigtes Gelkissen wird zwischen die Kontaktflächen, also zwischen die Zähne und den Ultraschallscanner, positioniert, um die Kopplung zu verbessern. Zum Auswerten der Aufnahmen schlägt die Arbeit eine Systemarchitektur vor, die auf maschinellem Lernen beruht und die zur automatischen Erkennung und Segmentierung des Alveolarknochens in Ultraschallbildern trainiert ist.

[0009] Limitationen bei dieser Arbeit ergeben sich durch die Qualität der Ultraschallaufnahmen und der Trainingsdaten. Insbesondere durch das freihändige Scannen der Zähne entstehen Einbußen in der Bildqualität sowie in der Vergleichbarkeit der Aufnahmen.

[0010] Aus dem Stand der Technik sind zudem Bohrhilfen bekannt. Bohrhilfen unterstützen bei einem exakten Anbringen eines Führungsloches für ein Zahnimplantat, wobei das Führungsloch für das Implantat an noch im Kiefer vorhandenen Zähnen ausgerichtet wird.

[0011] Aus DE 199 52 962 A1 ist beispielsweise ein Verfahren zur Herstellung einer Bohrhilfe für ein Zahnimplantat bekannt. Hierfür werden zunächst Röntgenaufnahmen des Kiefers und entsprechende Messdatensätze hergestellt. Danach wird eine dreidimensionale, optische Vermessung der sichtbaren Oberfläche von Kiefer und Zähnen durchgeführt sowie ein entsprechender Messdatensatz erzeugt. Die Messdatensätze von Röntgenaufnahme und die Messdatensätze der dreidimensionalen optischen Aufnahme werden miteinander korreliert. Durch die dann vorhandene Information wird die Art und Position des Implantates relativ zu den Nachbarzähnen geplant und eine Bohrschablone generiert, die auf den Nachbarzähnen befestigt wird und so die exakte Bohrung des Implantatführungsloches ermöglicht.

[0012] Aus dem Stand der Technik hingegen nicht bekannt sind Vorrichtungen, die dabei unterstützen, Zähne eines Patienten mit einem Ultraschallscanner bzw. Ultraschallkopf in einer reproduzierbaren Position zu scannen, bzw. Verfahren zum Herstellen derartiger Vorrichtungen.

[0013] Es ist daher eine Aufgabe der vorliegenden Erfindung eine Vorrichtung zum reproduzierbaren Positionieren eines Ultraschallkopfs anzugeben, die intraoral einsetzbar ist, und die eine standardisierte, nicht-invasive Bildgebung von einer intraoralen, anatomischen Struktur eines Patienten unterstützt.

[0014] Die Aufgabe wird durch die Merkmale der unabhängigen Patentansprüche gelöst. Bevorzugte Ausgestaltungen sind Gegenstand der abhängigen Ansprüche.

[0015] In einem ersten Aspekt der Erfindung wird die Aufgabe mit einer Vorrichtung für eine intraorale Positionierung eines Ultraschallkopfes nach Anspruch 1 gelöst. Die Vorrichtung umfasst eine Dentalschiene, die einen Dentalschienenkörper aufweist, der an und/oder auf einer intraoralen, anatomischen Struktur temporär positionierbar ist und dessen intraorale Positionierung durch die intraorale, anatomische Struktur definiert ist. Der Begriff der „Position“ umfasst auch eine Orientierung des Dentalschienenkörpers, die ebenfalls durch die Positionierung festgelegt ist. Der Dentalschienenkörper bildet die intraorale, anatomische Struktur derart nach, dass der Dentalschienenkörper in exakt einer räumlich definierten Position formschlüssig an bzw. auf die intraorale, anatomische Struktur positionierbar ist. Die Dentalschiene weist einen Aufnahmeabschnitt auf zum Aufnehmen eines Ultraschallkopfes mit optionalem Adapter und/oder Gelkissen. Der Aufnahmeabschnitt weist einen Schalldurchtrittsbereich, wie beispielsweise eine Öffnung oder einen Bereich mit schalleitendem Material auf, durch den Ultraschallwellen von dem Ultraschallkopf zu einem darzustellenden Abschnitt der intraoralen, anatomischen Struktur hindurchtreten können, und wenigstens ein Positionierelement, mit dem der Ultraschallkopf in Kontakt gebracht werden kann, um die Pose des Ultraschallkopfes relativ zur Dentalschiene zu definieren. Die Pose des Ultraschallkopfes umfasst sowohl seine örtliche Position als auch seine Orientierung in Bezug auf die Dentalschiene.

[0016] Durch das Vorsehen eines Dentalschienenkörpers, dessen intraorale Positionierung durch die intraorale, anatomische Struktur definiert ist und der in exakt einer räumlich definierten Position formschlüssig an bzw. auf der intraoralen, anatomischen Struktur positionierbar ist, ist die Vorrichtung eingerichtet, intraoral eingesetzt zu werden. Zudem soll verstanden werden, dass die intraorale, anatomische Struktur eine individuelle Struktur eines spezifischen Patienten ist, und dass die Vorrichtung insofern eine individuelle Vorrichtung ist.

[0017] Aufgrund des Schalldurchtrittsbereichs, wie insbesondere Öffnung, an dem Aufnahmeabschnitt zum Durchlassen von Ultraschallwellen ist die Vorrichtung geeignet, eine Bildgebung mittels Ultraschall zu ermöglichen. Die von dem Ultraschallkopf emittierten Ultraschallwellen werden also nicht an dem Aufnahmeabschnitt reflektiert, sondern können durch dessen Öffnung zu dem darzustellenden Abschnitt der intraoralen, anatomischen Struktur hindurchtreten und von diesem dann reflektiert werden. Es soll verstanden werden, dass der darzustellende Abschnitt der intraoralen, anatomischen Struktur insbesondere ein Implantationsbereich in der intraoralen, anatomischen Struktur ist, d.h. ein Bereich, in dem ein Implantat eingesetzt wurde und der sowohl Knochen als auch Weichgewebe umfasst.

[0018] Mit dem Positionierelement an dem Aufnahmeabschnitt kann die Pose des Ultraschallkopfes relativ zur Dentalschiene bestimmt werden und insofern relativ zur intraoralen, anatomischen Struktur, sofern die Dentalschiene intraoral positioniert ist. Folglich kann der Ultraschallkopf reproduzierbar ausgerichtet werden, sodass Aufnahmen des darzustellenden Abschnitts mittels Ultraschall auf standardisierte und vergleichbare Weise aufgenommen werden können. Veränderungen des Knochens und/oder des Weichgewebes an dem darzustellenden Bereich können somit zuverlässig und frühzeitig erkannt werden. Neben der Reproduzierbarkeit der Aufnahme ermöglicht die bekannte Lagebeziehung zwischen Ultraschallkopf und einem etwaigen Implantat die Messung der Knochendicke zum

Implantat. Eine Messung der Knochendicke wäre mittels Ultraschall allein ohne die erfindungsgemäße Vorrichtung nicht möglich, da Ultraschall im genutzten Frequenzbereich die Knochenoberfläche kaum durchdringt.

[0019] Es ist bevorzugt, dass das Positionierelement ein Rahmen ist, der formschlüssig zur Anlage mit dem Ultraschallkopf eingerichtet ist. Das Positionierelement kann alternativ oder zusätzlich ein Führungsmittel, eine Buchse und/oder einen Adapter aufweisen. Das Führungsmittel ist dann bevorzugt dazu eingerichtet, den Ultraschallkopf in die vorbestimmte Pose zu führen. Der Aufnahmeabschnitt ist bevorzugt dazu eingerichtet, einen vorbestimmten Ultraschallkopf aufzunehmen. Besonders bevorzugt ist, dass der Aufnahmeabschnitt dazu eingerichtet ist, unterschiedliche Ultraschallköpfe aufzunehmen. Ultraschallköpfe unterschiedlicher Anbieter bzw. Marken können in ihrer Form und/oder ihrer Größe voneinander abweichen. Zum Aufnehmen von unterschiedlichen Ultraschallköpfen ist es bevorzugt, dass in dem Aufnahmeabschnitt unterschiedliche Adapter positionierbar sind, wobei die unterschiedlichen Adapter dazu eingerichtet sind, unterschiedliche Ultraschallköpfe jeweils passgenau und formschlüssig aufzunehmen. Die Adapter weisen bevorzugt einen mit dem Schalldurchtrittsbereich des Aufnahmeabschnitts korrespondierenden Durchgang auf, sodass die Ultraschallwellen der jeweiligen Ultraschallköpfe auf den darzustellenden Abschnitt treffen können. Zum Aufnehmen von unterschiedlichen Ultraschallköpfen ist alternativ bevorzugt, dass der Aufnahmeabschnitt mit einer aushärtbaren Masse ausgekleidet werden kann, wobei die aushärtbare Masse an einen bevorzugten Ultraschallkopf formgenau angepasst werden kann.

[0020] Der Aufnahmeabschnitt ist bevorzugt dazu eingerichtet, ein mit einem Koppelmittel gefülltes Pad zwischen der intraoralen, anatomischen Struktur, insbesondere zwischen dem darzustellenden Abschnitt der intraoralen, anatomischen Struktur, und der Vorrichtung in Position zu halten. Hierzu kann der Aufnahmeabschnitt eine Haltevorrichtung aufweisen und/oder durch Klemmung das Pad in Position halten. Der Aufnahmeabschnitt hält das Pad vorzugsweise derart in Position, dass das Pad die Öffnung des Aufnahmeabschnitts im Wesentlichen vollständig abdeckt. Das Pad kann zum Verschließen der Öffnung auch direkt an der Öffnung des Aufnahmeabschnitts angeordnet sein. Vorzugsweise weist die Dentalschiene ein integriertes Pad auf, welches die Öffnung vorzugsweise vollständig bedeckt. Alternativ hierzu kann das mit Koppelmittel gefüllte Pad an den Ultraschallkopf befestigt sein, wobei der Aufnahmeabschnitt dann bevorzugt dazu eingerichtet ist, den Ultraschallkopf mitsamt dem Pad aufzunehmen, vorzugsweise derart, dass die Öffnung des Aufnahmeabschnitts im Wesentlichen vollständig von dem Pad bedeckt bzw. ausgefüllt ist.

[0021] In einer bevorzugten Weiterbildung weist der Aufnahmeabschnitt eine verschließbare Tasche auf, die die Öffnung des Aufnahmeabschnitts vorzugsweise vollständig abdeckt oder verschließt, und die mit Koppelmittel befüllbar oder befüllt ist. Die Tasche kann dann mit einem beliebigen Koppelmittel vor jedem Aufnahmevorgang befüllt werden und nach Beendigung des Aufnahmevorgangs entleert werden.

[0022] Durch das Koppelmittel wird die Übertragung der Ultraschallwellen verbessert, indem das Koppelmittel verhindert, dass sich zwischen dem Ultraschallkopf und dem darzustellenden Abschnitt der intraoralen, anatomischen Struktur ungewollte Einschlüsse, insbesondere Lufteinschlüsse, befinden. Das Koppelmittel besteht vorzugsweise aus einer viskosen Flüssigkeit oder einem Gel.

[0023] Der Dentalschienenkörper weist vorzugsweise einen Plattformabschnitt und einen Auflageabschnitt auf. Der Aufnahmeabschnitt zum Aufnehmen des Ultraschallkopfs ist vorzugsweise an dem Plattformabschnitt angeordnet und erstreckt sich vorzugsweise orthogonal von dem Plattformabschnitt. Der Auflageabschnitt ist vorzugweise in exakt einer räumlich definierten Position formschlüssig mit der intraoralen, anatomischen Struktur in Eingriff bringbar. Der Auflageabschnitt liegt insofern bevorzugt direkt auf der intraoralen, anatomischen Struktur auf. Es ist also bevorzugt, dass der Dentalschienenkörper wie eine Bissschiene an bzw. auf der intraoralen, anatomischen Struktur positionierbar ist. Vorzugsweise wird eine Geometrie der intraoralen, anatomischen Struktur mittels nicht-invasiven Mitteln aufgenommen, vorzugsweise mittels Volumentomographie, optischem Scanner (optical scan) oder mittels Ultraschall. An der aufgenommenen Geometrie werden anschließend Kontaktpunkte festgelegt. Der Dentalschienenkörper, insbesondere der Auflageabschnitt, weist vorzugsweise Anlagepunkte auf, die bei Positionierung des Dentalschienenkörpers an und/oder auf der intraoralen, anatomischen Struktur formschlüssig mit den festgelegten lagebestimmenden Kontaktpunkten oder Kontaktflächen in Kontakt treten.

[0024] In einer bevorzugten Weiterbildung wird der Auflageabschnitt durch eine Bohrschablone gebildet. Es soll verstanden werden, dass die Bohrschablone vorzugsweise eine Bohrschablone ist, die zum Fertigen einer Bohrung für ein Implantat in der intraoralen, anatomischen Struktur verwendet wurde. Die Bohrschablone weist vorzugsweise einen Unterbrechungsabschnitt auf zum Freigeben eines Implantationsbereichs, wenn die Vorrichtung intraoral positioniert ist. Der Plattformabschnitt ist vorzugsweise

ebenmäßig und/oder flach ausgebildet ist und liegt vorzugsweise auf der Bohrschablone auf.

[0025] Vorzugsweise ist die Bohrschablone mit dem Plattformabschnitt kraftschlüssig, formschlüssig und/oder stoffschlüssig verbunden. Beispielsweise ist die Bohrschablone mit dem Plattformabschnitt durch Kleben, Löten, Nieten, Schrauben und/oder Zusammensetzen verbunden. Vorzugsweise ist der Plattformabschnitt an einer äußeren Oberfläche der Bohrschablone befestigt. In einer besonders bevorzugten Ausführungsform sind die Bohrschablone und der Plattformabschnitt einstückig ausgebildet. Vorzugsweise sind die Bohrschablone und der Plattformabschnitt aus dem gleichen und/oder ähnlichen Material bzw. aus den gleichen und/oder ähnlichen Materialien ausgebildet.

[0026] Vorzugsweise weist die Bohrschablone ein erstes Formschlusselement und der Plattformabschnitt ein zweites Formschlusselement auf, wobei das zweite Formschlusselement mit dem ersten Formschlusselement zum formschlüssigen Festlegen der Verbindung von der Bohrschablone und dem Plattformabschnitt korrespondiert. Die Bohrschablone und der Plattformabschnitt sind insofern bevorzugt dazu ausgebildet, ineinander einzugreifen, um eine formschlüssige Verbindung einzugehen. Zusätzlich kann ein Verbindungselement für die formschlüssige Verbindung von der Bohrschablone und dem Plattformabschnitt vorgesehen sein. Beispielsweise kann das erste Formschlusselement ein oder mehrere Stifte sein, wobei das zweite Formschlusselement dann ein korrespondierendes Loch bzw. korrespondierende Löcher ist/sind. In diesem Fall kann der Plattformabschnitt formschlüssig auf die Bohrschablone gesteckt werden.

[0027] In einer bevorzugten Ausführungsform ist der Plattformabschnitt an wenigstens zwei Bohrpositionen an der Bohrschablone befestigt ist. Vorzugsweise sind die Bohrpositionen symmetrisch zu einer Symmetrieachse der Dentalschiene angeordnet. Vorzugsweise ist der Aufnahmeabschnitt exakt zwischen den Bohrpositionen angeordnet. Es können auch mehr als zwei Bohrpositionen vorgesehen sein, beispielsweise vier, fünf oder sechs, wobei die Bohrpositionen vorzugsweise symmetrisch zu der Symmetrieachse der Dentalschiene angeordnet sind.

[0028] Insbesondere ist bevorzugt, dass die Bohrschablone eine Aussparung aufweist, wobei der Plattformabschnitt vorzugsweise formschlüssig, stoffschlüssig und/oder kraftschlüssig in der Aussparung aufgenommen ist, vorzugsweise derart aufgenommen ist, dass die Lage des Plattformabschnitts (und damit des Ultraschallkopfes) reproduzierbar ist. Dies stellt eine besonders einfache und sichere Verbindung dar.

[0029] In einer bevorzugten Weiterbildung weist der Dentalschienenkörper wenigstens einen Stift und der Aufnahmeabschnitt wenigstens eine Bohrung auf, durch die sich der Stift erstreckt, zum relativen Positionieren des Dentalschienenkörpers und des Aufnahmeabschnitts zueinander. Dies ermöglicht ein Einstellen des Abstands zwischen dem Ultraschallkopf und der anatomischen Struktur und erweitert so die Flexibilität. Der wenigsten eine Stift muss keinen zylindrischen Querschnitt haben, sondern kann jegliche Querschnittsform haben, etwa oval, rechteckig oder mehreckig.

[0030] In einem zweiten Aspekt der Erfindung wird das Problem durch ein Verfahren zum Herstellen einer Vorrichtung für die reproduzierbare intraorale Positionierung eines Ultraschallkopfes gelöst. Das Verfahren umfasst den Schritt eines Bereitstellens einer Bohrschablone, die in exakt einer räumlich definierten Position formschlüssig mit einer intraoralen, anatomischen Struktur in Eingriff bringbar ist, und die eine Bohrführung aufweist zur Führung eines Bohrers zum Fertigen einer Bohrung für ein Implantat in einer intraoralen, anatomischen Struktur. Mithin soll vorzugsweise eine Bohrschablone bereitgestellt werden, die zum Einsetzen eines Implantats in der intraoralen, anatomischen Struktur verwendet wurde. Zudem umfasst das Verfahren ein Bereitstellen eines Plattformabschnitts, an dem ein Aufnahmeabschnitt zum Aufnehmen eines Ultraschallkopfes angeordnet ist, wobei der Aufnahmeabschnitt einen Schalldurchtrittsbereich, vorzugsweise eine Öffnung, aufweist, durch den Ultraschallwellen von dem Ultraschallkopf hindurchtreten können, und wobei der Aufnahmeabschnitt wenigstens ein Positionierelement aufweist, mit dem der Ultraschallkopf in Kontakt gebracht werden kann. Das Verfahren umfasst zudem ein Verbinden der Bohrschablone mit dem Plattformabschnitt, wobei bei der Bohrschablone ein Kronenabschnitt entfernt wird, wobei der Kronenabschnitt ein Abschnitt der Bohrschablone ist, der bei intraoraler Positionierung an und/oder auf einer Krone eines in der intraoralen, anatomischen Struktur eingesetzten Implantats liegen würde, und wobei der Kronenabschnitt die Bohrführung umfasst. Die Bohrschablone wird vorzugsweise formschlüssig, kraftschlüssig und/oder stoffschlüssig mit dem Plattformabschnitt verbunden. Der Plattformabschnitt wird vorzugsweise derart mit der Bohrschablone verbunden, dass eine Symmetrieachse des Aufnahmeabschnitts parallel zu einer Mittelachse der Bohrführung ist, sodass die Mittelachse der Bohrführung in der Bildebene des Ultraschallkopfes liegt.

[0031] Wird eine zuvor bestehende Bohrschablone getrennt, um Plattformabschnitt einzusetzen, sollte die Lagebeziehung der beiden Teile der Bohrschablone beibehalten bleiben. Die Lagebeziehung zwischen Bohrschablone und Plattformabschnitt muss bekannt sein.

[0032] Es soll verstanden werden, dass die Bohrschablone vorzugsweise basierend auf nicht-invasiven Aufnahmen oder einer plastischen Rekonstruktion einer Geometrie einer intraoralen, anatomischen Struktur eines Patienten gefertigt wurde. Ferner soll verstanden werden, dass die Bohrschablone vorzugsweise Anlagepunkte aufweist, die bei intraoraler Positionierung formschlüssig mit Kontaktpunkten der intraoralen, anatomischen Struktur in Kontakt treten.

[0033] Es soll verstanden werden, dass die Bohrschablone und der Plattformabschnitt mitsamt dem Aufnahmeabschnitt auch integral bzw. einstückig hergestellt werden können. Hierzu ist bevorzugt, dass ein integrierter Datensatz basierend auf nicht-invasiven Aufnahmen einer Geometrie einer intraoralen, anatomischen Struktur eines Patienten erzeugt wird, der eine integrierte Ausführung einer Bohrschablone und eines Plattformabschnitts mitsamt dem Aufnahmeabschnitt umfasst. Dieser integrierte Datensatz wird dann vorzugsweise an ein CAM-System übertragen. Mit dem CAM-System wird ein Maschinenprogramm, beispielsweise ein NC-Programm, erzeugt für die Herstellung der integrierten Vorrichtung. Zum Herstellen der integrierten Vorrichtung wird das Maschinenprogramm dann ausgeführt.

[0034] Möglich ist zudem, dass zum Herstellen einer Vorrichtung für die intraorale Positionierung eines Ultraschallkopfes eine intraorale, anatomische Struktur plastisch abgeformt wird (Zahnabdruck), wobei die intraorale, anatomische Struktur vorzugsweise wenigstens einen Implantationsbereich mit vorzugsweise einem Implantat und/oder einer Krone umfasst. Bevorzugt wird anschließend basierend auf der plastischen Abformung bzw. dem Abdruck die intraorale, anatomische Struktur durch ein Rekonstruktionsmodell rekonstruiert. Abschließend wird vorzugsweise eine Dentalschiene hergestellt, die einen Dentalschienenkörper und einen Aufnahmeabschnitt aufweist, wobei der Dentalschienenkörper an das Rekonstruktionsmodell angepasst ist, sodass er dazu eingerichtet ist, an und/oder auf der intraoralen, anatomischen Struktur positioniert zu werden. Vorzugsweise wird eine zusätzliche Registrierung des Zahnabdrucks beispielsweise mit einem Abformpfosten, vorgenommen, um eine Lagebeziehung zwischen dem Aufnahmeabschnitt zu definieren. Der Aufnahmeabschnitt ist vorzugsweise zur Aufnahme eines Ultraschallkopfes eingerichtet und weist vorzugsweise einen Schalldurchtrittsbereich, vorzugsweise eine Öffnung, sowie wenigstens ein Positionierelement zur definierten Positionierung des Ultraschallkopfes relativ zur Dentalschiene auf. Der Aufnahmeabschnitt wird vorzugweise derart an der Dentalschiene angeordnet, dass die Öffnung des Aufnahmeabschnitts auf Höhe des Implantationsbereichs angeordnet ist und diesen wenigstens teilweise freigibt.

[0035] In einem dritten Aspekt der Erfindung wird das Problem durch eine Verwendung einer Vorrichtung für eine intraorale Positionierung eines Ultraschallkopfes, vorzugsweise wie vorstehend beschrieben, zum Aufnehmen von intraoralen Ultraschallbilden eines darzustellenden Abschnitts einer intraoralen, anatomischen Struktur mit einem Ultraschallkopf, gelöst.

[0036] Zum Verwenden der Vorrichtung wird vorzugsweise ein Dentalschienenkörper einer Dentalschiene an und/oder auf der intraoralen, anatomischen Struktur positioniert, wobei die Positionierung des Dentalschienenkörpers durch die intraorale, anatomische Struktur definiert ist, und wobei der Dentalschienenkörper in exakt einer räumlich definierten Position formschlüssig an und/oder auf die intraorale, anatomische Struktur positionierbar ist. Die zu verwendende Vorrichtung weist vorzugsweise einen Aufnahmeabschnitt zum Aufnehmen eines Ultraschallkopfes auf, wobei der Aufnahmeabschnitt eine Öffnung und ein Positionierelement aufweist.

[0037] Der Ultraschallkopf wird vorzugsweise mit dem Positionierelement des Aufnahmeabschnitts in Kontakt gebracht zum Positionieren des Ultraschallkopfes relativ zur Dentalschiene. Ein mit einem Koppelmittel gefülltes Pad wird zwischen den darzustellenden Abschnitt der intraoralen, anatomischen Struktur und den Ultraschallkopf, vorzugsweise zwischen den darzustellenden Abschnitt der intraoralen, anatomischen Struktur und den Aufnahmeabschnitt der Dentalschiene positioniert. Es soll verstanden werden, dass die Reihenfolge dieser Schritte nicht festgelegt ist. Es kann also sowohl zunächst der Ultraschallkopf mit dem Positionierelement in Kontakt gebracht werden und anschließend das Pad positioniert werden, aber auch zunächst das Pad positioniert werden und anschließend der Ultraschallkopf mit dem Positionierelement in Kontakt gebracht werden.

[0038] Alternativ hierzu kann das mit Koppelmittel gefüllte Pad auch an den Ultraschallkopf befestigt werden, wobei der Aufnahmeabschnitt dann bevorzugt dazu eingerichtet ist, den Ultraschallkopf mitsamt dem Pad aufzunehmen, vorzugsweise derart, dass die Öffnung des Aufnahmeabschnitts vollständig von dem Pad bedeckt bzw. ausgefüllt ist. Das Positionieren des Pads und das in Kontakt bringen des Ultraschallkopfes mit dem Positionierelement findet dann vorzugsweise zeitgleich statt. In einer weiteren bevorzugten Alternative wird eine verschließbare Tasche der Dentalschiene, die die Öffnung des Aufnahmeabschnitts vorzugsweise vollständig abdeckt oder verschließt, mit Koppelmittel befüllt. In einer weiteren Alternative wird, für den Fall, dass die Dentalschiene ein integriertes Pad aufweist, kein zusätzliches Pad positioniert.

[0039] Mit dem Ultraschallkopf werden dann Ultraschallbilder durch Senden und Empfangen von Ultraschallwellen aufgenommen, die durch den Schalldurchtrittsbereich, vorzugsweise die Öffnung, des Aufnahmeabschnitts zu dem darzustellenden Abschnitt der intraoralen, anatomischen Struktur hindurchtreten. Es soll verstanden werden, dass der darzustellende Abschnitt vorzugsweise ein Implantationsbereich mit einem Implantat und einer Krone ist, wobei der Implantationsbereich sowohl Knochen (bukkaler Knochen) als auch Weichgebe aufweist. Vorzugsweise werden Ultraschallbilder aufgenommen, die sowohl das Weichgebe als auch den Knochen des Implantationsbereichs darstellen.

[0040] Vorzugsweise wird die Vorrichtung für die intraorale Positionierung des Ultraschallkopfes für eine Verlaufskontrolle verwendet. Hierfür werden vorzugsweise intraorale Ultraschallbilder in vorbestimmten Zeitabständen aufgenommen, wobei der Ultraschallkopf für die Aufnahmen stets mit dem Positionierelement des Aufnahmeabschnitts in Kontakt gebracht wird. Der Zeitabstand zwischen den Aufnahmen kann bspw. eine Woche, ein Monat, mehrere Monate, ein Jahr oder ein sonstig beliebiges Zeitintervall sein. Durch das Aufnehmen von Ultraschallbildern in vorbestimmten Zeitabständen können Veränderungen des darzustellenden Abschnitts, insbesondere Veränderungen des Knochens und/oder des Weichgewebes des darzustellenden Abschnitts, frühzeitig erkannt werden. Dadurch, dass der Ultraschallkopf stets mit dem Positionierelement des Aufnahmeabschnitts in Kontakt gebracht wird, wird der Ultraschallkopfs reproduzierbar ausgerichtet, wodurch eine hohe Vergleichbarkeit der Aufnahmen gewährleistet ist. Neben der Reproduzierbarkeit der Aufnahme ermöglicht die bekannte Lagebeziehung zwischen Ultraschallkopf und einem etwaigen Implantat die Messung der Knochendicke zum Implantat. Eine Messung der Knochendicke wäre mittels Ultraschall allein ohne die erfindungsgemäße Vorrichtung nicht möglich, da Ultraschall im genutzten Frequenzbereich die Knochenoberfläche kaum durchdringt.

[0041] Ausführungsformen der Erfindung werden nun nachfolgend anhand der Zeichnungen beschrieben. Diese sollen die Ausführungsformen nicht notwendigerweise maßstäblich darstellen, vielmehr sind die Zeichnungen, wenn dies zur Erläuterung dienlich ist, in schematisierter und/oder leicht verzerrter Form ausgeführt. Im Hinblick auf Ergänzungen der aus den Zeichnungen unmittelbar erkennbaren Lehren wird auf den einschlägigen Stand der Technik verwiesen. Dabei ist zu berücksichtigen, dass vielfältige Modifikationen und Änderungen betreffend die Form und das Detail einer Ausführungsform vorgenommen werden können, ohne von der allgemeinen Idee der Erfindung abzuweichen. Die in der Beschreibung, in den Zeichnungen sowie in den Ansprüchen offenbarten Merkmale der Erfindung können sowohl einzeln als auch in beliebiger Kombination für die Weiterbildung der Erfindung wesentlich sein. Zudem fallen in den Rahmen der Erfindung alle Kombinationen aus zumindest zwei der in der Beschreibung, den Zeichnungen und/oder den Ansprüchen offenbarten Merkmale. Die allgemeine Idee der Erfindung ist nicht beschränkt auf die exakte Form oder das Detail der im Folgenden gezeigten und beschriebenen bevorzugten Ausführungsformen oder beschränkt auf einen Gegenstand, der eingeschränkt wäre im Vergleich zu dem in den Ansprüchen beanspruchten Gegenstand. Bei angegebenen Bemessungsbereichen sollen auch innerhalb der genannten Grenzen liegende Werte als Grenzwerte offenbart und beliebig einsetzbar und beanspruchbar sein. Der Einfachheit halber sind nachfolgend für identische oder ähnliche Teile oder Teile mit identischer oder ähnlicher Funktion gleiche Bezugszeichen verwendet.

[0042] Weitere Vorteile, Merkmale und Einzelheiten der Erfindung ergeben sich aus der nachfolgenden Beschreibung der bevorzugten Ausführungsformen sowie anhand der Zeichnungen; diese zeigen in:

Fig. 1 eine perspektivische Darstellung eines ersten Ausführungsbeispiels der Vorrichtung;

Fig. 2 die Anordnung aus **Fig.** 1 in Explosionsdarstellung;

Fig. 3 eine perspektivische Darstellung des ersten Ausführungsbeispiels der Vorrichtung mit positioniertem Ultraschallkopf;

Fig. 4 eine teilweise geschnittene Darstellung einer intraoralen, anatomischen Struktur mit positionierter Vorrichtung gemäß dem ersten Ausführungsbeispiel;

Fig. 5a, 5b zwei perspektivische Ansichten des Plattformabschnitts;

Fig. 6 ein Flussdiagramm zur Veranschaulichung eines bevorzugten Verfahrens zum Herstellen einer Vorrichtung für die intraorale Positionierung eines Ultraschallkopfes;

Fig. 7 ein Flussdiagramm zur Veranschaulichung eines alternativen Verfahrens zum Herstellen einer Vorrichtung für die intraorale Positionierung eines Ultraschallkopfes; und in

Fig. 8 bis 10 ein weiteres Ausführungsbeispiel der Vorrichtung.

[0043] **Fig.** 1 zeigt eine schematische Darstellung eines menschlichen Unterkiefers 50. Der Unterkiefer 50 umfasst Knochen 52, der von Weichgebe 54 bedeckt ist (vgl. **Fig.** 4). Ein Zahnbogen 56 erstreckt sich entlang des Unterkiefers 50, wobei insbesondere die Zahnkronen 58 zu erkennen sind. Der Zahnbogen 56 umfasst einen Implantationsbereich 60,

d.h. einen Bereich mit einem eingesetzten Implantat 62 und einer Krone 64 (vgl. **Fig.** 4). Das Implantat 62 ist in dem Knochen 52 (bukkaler Knochen) verankert (vgl. **Fig.** 4). Gemäß **Fig.** 1 definiert der Zahnbogen 56 und das Weichgebe 54 eine intraorale, anatomische Struktur 8.

[0044] Eine Vorrichtung 1 für eine intraorale Positionierung eines Ultraschallkopfes 2 ist gemäß **Fig.** 1 an und auf der intraoralen, anatomischen Struktur 8 angeordnet. Die Vorrichtung 1 umfasst eine Dentalschiene 4 mit einem Dentalschienenkörper 6 und einem Aufnahmeabschnitt 10. Der Dentalschienenkörper weist einen Plattformabschnitt 5 und einen Auflageabschnitt 7 auf. Der Dentalschienenkörper 6, insbesondere der Auflageabschnitt 7, ist auf der intraoralen, anatomischen Struktur 8, insbesondere auf den Zahnkronen 58 des Zahnbogens 56 positioniert. Der Aufnahmeabschnitt 10 ist an dem Dentalschienenkörper 6, insbesondere an dem Plattformabschnitt 5 angeordnet. Gemäß **Fig.** 1 ist der Plattformabschnitt 5 flächig und rechteckig ausgebildet, sodass er flach auf dem Auflageabschnitt 7 aufliegt. Selbstverständlich sind auch andere Formen möglich. Wichtig ist, dass der Plattformabschnitt 5 in definierter Lage an dem Auflageabschnitt 7 angeordnet ist, vorzugsweise in allen sechs Freiheitsgraden definiert, beispielsweise über sechs eindeutige Auflagepunkte und/oder Flächen. Die Positionierung des Dentalschienenkörpers 6 ist durch die intraorale, anatomische Struktur 8, insbesondere durch den Zahnbogen 56, definiert.

[0045] Der Aufnahmeabschnitt 10 ist im Wesentlichen orthogonal zu dem Plattformabschnitt 5 ausgerichtet. Der Aufnahmeabschnitt 10 liegt vorzugsweise wenigstens teilweise an Weichgewebe 54 der intraoralen, anatomischen Struktur 8 an (vgl. **Fig.** 4). Der Aufnahmeabschnitt 10 ist dazu eingerichtet, einen Ultraschallkopf 2, insbesondere einen vorbestimmten Ultraschallkopf 2, aufzunehmen. **Fig.** 3 zeigt, dass ein Ultraschallkopf 2 von bzw. in dem Aufnahmeabschnitt 10 aufgenommen ist. Der Aufnahmeabschnitt 10 weist einen Schalldurchtrittsbereich 12 in Form einer Öffnung 12 auf, durch die Ultraschallwellen von dem Ultraschallkopf 2 zu einem darzustellenden Abschnitt 14 der intraoralen, anatomischen Struktur 8 hindurchtreten können. In der Öffnung 12 ist ein Gelkissen 13 angeordnet. Vorliegend ist der darzustellende Abschnitt 14 der Implantationsbereich 60 im Zahnbogen 56. Der Implantationsbereich 60 umfasst sowohl Weichgebe 54 als auch Knochen 52 (bukkaler Knochen) in den das Implantat 62 eingesetzt ist (vgl. **Fig.** 4).

[0046] Der Aufnahmeabschnitt 10 weist zudem ein Positionierelement 16 auf, mit dem der Ultraschallkopf 2 in Kontakt gebracht werden kann, um die Positionierung des Ultraschallkopfes 2 relativ zur Dentalschiene 4 zu definieren. Das Positionierelement 16 ist gemäß **Fig.** 1 als Rahmen 18 ausgebildet, welcher die Öffnung 12 des Aufnahmeabschnitts 10 umgibt. Der Rahmen 18 weist zudem Rahmenwände 19 auf, die eine gewisse Tiefe aufweisen. Insofern ist der Rahmen 18 geeignet, den Ultraschallkopf 2 in eine endgültige bzw. vorbestimmte Position zu führen. Dadurch, dass der Rahmen 18 eine gewisse Tiefe aufweist, kann der Ultraschallkopf 2 in der vorbestimmten Position stabilisiert werden.

[0047] Gemäß **Fig.** 1 ist der Auflageabschnitt 7 als Bohrschablone 22 ausgebildet. Die Bohrschablone 22 bildet die intraorale, anatomische Struktur 8, insbesondere den Zahnbogen 56 des Unterkiefers 50 derart nach, dass die Bohrschablone 22 in exakt einer räumlich definierten Position formschlüssig auf der intraoralen, anatomischen Struktur 8, insbesondere auf dem Zahnbogen 56 positioniert ist. Die Bohrschablone 22 ist bogenförmig ausgebildet und bedeckt einen Großteil des Zahnbogens 56. Es soll verstanden werden, dass die Bohrschablone 22 auch den gesamten Zahnbogen 56 oder nur einen gewissen Bereich des Zahnbogens 56 bedecken kann. Zum formschlüssigen Positionieren der Bohrschablone 22 weist die Bohrschablone 22 Anlagepunkte 68 auf, die bei Einsetzen bzw. Positionieren der Vorrichtung 1 formschlüssig mit an der intraoralen, anatomischen Struktur 8, insbesondere dem Zahnbogen 56, festgelegten Kontaktpunkten 70 in Kontakt treten (vgl. **Fig.** 4).

[0048] Der Plattformabschnitt 5 weist zwei Bohrpositionen 24a, 24b auf, über die der Plattformabschnitt 5 an der Bohrschablone 22 befestigt werden kann. Der Plattformabschnitt 5 kann auch andere Formschlusselemente aufweisen, wobei verstanden werden soll, dass die Bohrschablone 22 dann korrespondierende Formschlusselemente bzw. Gegenstücke aufweist (nicht gezeigt). Ferner weist die Bohrschablone 22 eine Aussparung 26 auf, in die der Plattformabschnitt 5 eingesetzt ist. Aus **Fig.** 4 ist zudem ersichtlich, dass der Plattformabschnitt 5 eine Hakenstruktur 72 aufweist zum Fixieren des Plattformabschnitts 5 an der Bohrschablone 22. Insofern sind zum Verbinden des Plattformabschnitts 5 und der Bohrschablone 22 mehrere Verbindungseinrichtungen vorgesehen. Es soll verstanden werden, dass zum Verbinden des Plattformabschnitts 5 und der Bohrschablone 22 aber auch nur eine Verbindungseinrichtung, bspw. nur die Bohrpositionen 24a, 24b oder nur die Hakenstruktur 72, ausreichen kann. Darüber hinaus weist der Plattformabschnitt 5 eine Durchgangsbohrung 74 mit einer Durchgangsachse 76 auf, wobei die Durchgangsachse 76 koaxial mit einer Implantatachse 78 des eingesetzten Implantats 62 ist (vgl. auch **Fig.** 4). Die Durchgangsbohrung 74 hat vorzugsweise den Zweck, den Plattformabschnitt 5 in definierter, bekannter Lagebeziehung zur Bohrschablone 22 anzubringen, indem sie koaxial mit der Bohrachse ausgerichtet wird.

[0049] Es soll verstanden werden, dass die Bohrschablone 22 vorzugsweise eine Bohrschablone ist, die zum Fertigen einer Bohrung für das Implantat 62 in der intraoralen, anatomischen Struktur 8 verwendet wurde. Hierzu wies die Borschablone 22 vorzugsweise eine Bohrführung mit einer Mittelachse auf, die zur Führung des Bohrers zum Fertigen der Bohrung für das Implantat 62 in der intraoralen, anatomischen Struktur 8 eingerichtet war (nicht gezeigt). Es soll verstanden werden, dass eine derartige Mittelachse koaxial mit der Implantatachse 78 ist.

[0050] Da die Vorrichtung 1 jedoch nicht mehr zum Fertigen einer Bohrung für das Implantat 62 in der intraoralen, anatomischen Struktur 8 verwendet werden soll (dieses ist für den vorliegenden Anwendungsfall bereits eingesetzt), wurde ein Kronenabschnitt der Bohrschablone 22 entfernt, wobei der Kronenabschnitt ein Abschnitt der Bohrschablone 22 ist, der bei intraoraler Positionierung der Vorrichtung auf der Krone 64 des in der intraoralen, anatomischen Struktur 8 eingesetzten Implantats 62 liegen würde. In **Fig.** 1 weist die Bohrschablone 22 daher einen Unterbrechungsabschnitt 80 auf, sodass der Implantationsbereich 60 von der Borschablone 22 nicht bedeckt ist.

[0051] In den **Fig.** 1-4 ist eine Ultraschallebene 82 eingezeichnet, in der die Implantatachse 78 liegt. In der Ultraschallebene 82 liegt zudem eine Aufnahmeabschnittssymmetrieachse 84 des Aufnahmeabschnitts 10. Die Implantatachse 78 und die Aufnahmeabschnittsymmetrieachse 84 sind vorzugsweise parallel zueinander angeordnet. Der Aufnahmeabschnitt 10 ist im Wesentlichen parallel zu der Aufnahmeabschnittsymmetrieachse 84 ausgerichtet. Alternativ oder zusätzlich liegt die Implantatsachse in der Bildebene. Alternativ oder zusätzlich ist die Sondenachse 86, und entsprechend die Ausrichtung des Plattformabschnitts 5 und Bildachse, parallel zu einer Knochenoberfläche/Zahnfleischoberfläche ausgerichtet, um die Bildgebung der Knochenoberfläche zu verbessern und die Ankopplung durch z.B. ein Gel-Pad zu vereinfachen. Aus **Fig.** 4 geht zudem hervor, dass eine Ultraschallkopfachse 86 vorzugsweise parallel zu der Aufnahmeabschnittsymmetrieachse 84 und der Implantatachse 78 angeordnet ist, insbesondere wenn der Ultraschallkopf 2 in dem Aufnahmeabschnitt 10 aufgenommen ist (vgl. **Fig.** 3).

[0052] Wenn nun also der Ultraschallkopf 2 in dem Aufnahmeabschnitt 10 aufgenommen und positioniert ist, kann der Implantationsbereich 60 optimal untersucht werden (vgl. **Fig.** 3). Insbesondere können der Knochen 52 (bukkale Knochen), in den das Implantat 62 eingesetzt ist, und das Weichgewebe 54, welches das Implantat 62 umgibt, untersucht werden. Der Aufnahmeabschnitt 10 überragt insofern die Krone 64 des Implantats 62 und liegt an dem Weichgebe 54 wenigstens teilweise an, sodass durch die Öffnung 12 Ultraschallwellen zu dem Weichgebe 54 und dem bukkalen Knochen 52 gelangen können (**Fig.** 4).

[0053] **Fig.** 4 zeigt, dass der Aufnahmeabschnitt 10 zum Anliegen an dem Weichgewebe 54 einen Anlageabschnitt 88 aufweist. Der Anlageabschnitt 88 ist an einem unteren Rahmenabschnitt 90 des Rahmens 18 des Aufnahmeabschnitts 10 angeordnet. Es soll verstanden werden, dass die Dentalschiene 4 dazu eingerichtet ist, den Anlageabschnitt 88 auch bei sich verändernder Weichgewebsdicke an das Weichgewebe 54 anzulegen. Hierfür kann beispielsweise ein Verbindungsabschnitt 92 zwischen dem Plattformabschnitt 5 und dem Aufnahmeabschnitt 10 verformbar ausgebildet sein. Alternativ oder zusätzlich hierzu kann auch der Anlageabschnitt 88 verformbar ausgebildet sein.

[0054] **Fig.** 4 zeigt, dass der Aufnahmeabschnitt 10 dazu eingerichtet ist, ein mit einem Koppelmittel gefülltes Pad 20 zwischen der intraoralen, anatomischen Struktur 8, insbesondere zwischen dem darzustellenden Abschnitt 14 der intraoralen, anatomischen Struktur 8, und der Vorrichtung 1 in Position zu halten. Das mit dem Koppelmittel gefüllte Pad 20 ist vorzugsweise mit der Dentalschiene 4 verbunden. Das Pad 20 kann fest, aber auch lösbar mit der Dentalschiene 4 verbunden sein. Wenn die Vorrichtung 1 intraoral positioniert ist, ist das Pad 20 zwischen dem darzustellenden Abschnitt 14 und dem Aufnahmeabschnitt 10 angeordnet und bedeckt die Öffnung 12 des Aufnahmeabschnitts 10 an einem Ausgangsabschnitt 94 des Rahmens 18 vollständig. Alternativ hierzu kann die Dentalschiene 4 auch eine verschließbare Tasche aufweisen, die mit Koppelmittel befüllt werden kann (nicht gezeigt). Durch das Koppelmittel können Lufteinschlüsse zwischen dem Ultraschallkopf 2 und dem darzustellenden Abschnitt 14 vermieden werden, wodurch die Übertragung der Ultraschallwellen bzw. der Ultraschallenergie verbessert wird.

[0055] Zum Aufnehmen von intraoralen Ultraschallbilden des darzustellenden Abschnitts 14, insbesondere des Implantationsbereichs 60, welcher Knochen 52 und Weichgewebe 54 aufweist, wird der Ultraschallkopf 2 mit dem Positionierelement 16 bzw. dem Rahmen 18 des Aufnahmeabschnitts 10 in Kontakt gebracht. Zunächst wird der Ultraschallkopf 2 mit einem Eingangsabschnitt 96 des Rahmens 18 in Kontakt gebracht und durch die Rahmenwände 19 in eine vorbestimmte Position geführt. Wenn der Ultraschallkopf 2 in der vorbestimmten Position ist, ist er relativ zur Dentalschiene 4 und damit relativ zur intraoralen, anatomischen Struktur 8 positioniert (vgl. **Fig.** 3). **Fig.** 3 zeigt, dass der Ultraschalkopf 2 derart positioniert ist, dass die Implantatachse 78 des Implantats 62 in der Ultraschallebene 82 des Ultraschallkopfes 2 liegt. Durch die Rahmenwände 19

wird der Ultraschallkopf 2 in seiner Position stabilisiert, sodass dieser nicht verrutschen kann.

[0056] Durch die Vorrichtung 1 kann der Ultraschallkopf 2 reproduzierbar relativ zur Dentalschiene 4 und damit relativ zum Implantationsbereich 60 positioniert werden. Die hierdurch aufgenommenen Aufnahmen können zuverlässig miteinander verglichen und es können frühzeitig Veränderungen im Implantationsbereich erkannt werden. Außerdem ermöglicht die Registrierung und mithin die bekannte Transformationskette die Messung bukkaler Knochenoberflächen und Knochendicken zu einem Implantat.

[0057] Die **Fig.** 5a, 5b zeigen nochmal den Plattformabschnitt 5 samt Aufnahmeabschnitt 10 in zwei verschiedenen Perspektiven. In **Fig.** 5a ist auf der der Dentalstruktur zugewandten Seite des Plattformabschnitts 5 eine Vertiefung in Form einer Ausnehmung 150 zu sehen, in die ein Klebstoff aufgenommen werden kann. Ferner ist auf der der Dentalstruktur zugewandten Seite des Aufnahmeabschnitts 10 eine Vertiefung 152 vorgesehen, in die ein Gelpad 13 aufgenommen werden kann (in **Fig.** 5a nicht gezeigt).

[0058] Das Flussdiagramm in **Fig.** 6 veranschaulicht ein bevorzugtes Verfahren 100 zum Herstellen einer Vorrichtung für eine intraorale Positionierung eines Ultraschallkopfes. In einem ersten Schritt 102 wird eine Bohrschablone bereitgestellt, die in exakt einer räumlich definierten Position formschlüssig mit einer intraoralen, anatomischen Struktur in Eingriff bringbar ist. Es soll verstanden werden, dass die Bohrschablone zum Einsetzen eines Implantats in eine intraorale, anatomische Struktur eines Patienten verwendet wurde, dessen intraorale, anatomische Struktur nun für eine Verlaufskontrolle mittels Ultraschall untersucht werden soll. Die Bohrschablone weist insofern eine Bohrführung auf zur Führung eines Bohrers zum Fertigen einer Bohrung für ein Implantat in einer intraoralen, anatomischen Struktur.

[0059] Zudem wird ein Plattformabschnitt mit einem Aufnahmeabschnitt zum Aufnehmen des Ultraschallkopfes bereitgestellt 104. Der Aufnahmeabschnitt erstreckt sich vorzugsweise orthogonal zum dem Plattformabschnitt. Der Plattformabschnitt mit dem Aufnahmeabschnitt kann beispielsweise ein Spritzgussteil, gefräst oder mittels Additiver Fertigung hergestellt worden sein.

[0060] Die Bohrschablone wird mit dem Pattformabschnitt verbunden 106. Die Bohrschablone und der Plattformabschnitt werden derart miteinander verbunden, dass die Bohrschablone und der Plattformabschnitt einen Dentalschienenkörper einer Dentalschiene bilden. Bei der Bohrschablone wird entweder bevor die Bohrschablone mit dem Plattformabschnitt verbunden wird oder nachdem die Bohrschablone mit dem Plattformabschnitt verbunden wurde ein Kronenabschnitt entfernt. Der Kronenabschnitt ist ein Abschnitt der Bohrschablone ist, der bei intraoraler Positionierung an und/oder auf einer Krone eines in der intraoralen, anatomischen Struktur eingesetzten Implantats liegen würde. Wenn der Kronenabschnitt entfernt wurde weist die Bohrschablone einen Unterbrechungsabschnitt auf.

[0061] In einem alternativen Verfahren 110, welches in **Fig.** 7 gezeigt ist, wird von einem Patienten 112 ein Abdruck von einer intraoralen, anatomischen Struktur genommen 114. Die intraorale, anatomische Struktur weist vorzugsweise bereits wenigstens ein eingesetztes Implantat auf. Durch den Abdruck kann die aufgenommene intraorale, anatomische Struktur rekonstruiert werden, bspw. durch eine Gips-Rekonstruktion 116. Anhand der Rekonstruktion kann eine Dentalschiene passgenau für die intraorale, anatomische Struktur des Patienten gefertigt werden und deren Positionierung an oder auf der intraoralen, anatomischen Struktur definiert werden 118. Auch diese kann bei dem Patienten 112 eingesetzt werden, um reproduzierbare Ultraschallbilder aufzunehmen.

[0062] Die **Fig.** 8 bis 10 illustrieren eine Modifikation des Plattformabschnitts 5 und des Aufnahmeabschnitts 10. Während in dem in den **Fig.** 5a, 5b gezeigten Ausführungsbeispiel der Plattformabschnitt 5 und der Aufnahmeabschnitt 10 einstückig ausgebildet sind, sind sie in dem Ausführungsbeispiel der **Fig.** 8 bis 10 getrennt voneinander. Der Plattformabschnitt 5 ist hier vorzugsweise einstückig mit dem übrigen Dentalschienenkörper 6, auch wenn dies nicht zwingend ist. Auch könnte der Plattformabschnitt 5 in der Ausführungsform der **Fig.** 8 bis 10 entfallen und der Aufnahmeabschnitt 10 direkt mit dem übrigen Dentalschienenkörper 6 gekoppelt werden, wie sich aus der nachfolgenden Beschreibung ergeben wird.

[0063] Die Dentalschiene 4, vorzugsweise der Plattformabschnitt 5 und/oder der Dentalschienenkörper 6 weisen in dem hier gezeigten Ausführungsbeispiel einen ersten Stift 202 und einen zweiten Stift 204 auf (vgl. **Fig.** 9, 10). Die Stifte 202, 204 sind hier im Wesentlichen parallel zu einander und erstrecken sich weg von der dentalen Struktur, die aufgenommen werden soll.

[0064] Der Aufnahmeabschnitt 10 hat eine erste Bohrung 206 und eine zweite Bohrung 208, durch die sich die ersten und zweite Stifte 202, 204 erstrecken. Der Aufnahmeabschnitt 10 kann auf diese Weise auf den Stiften 202, 204 verschoben werden, und so der Abstand vom Ultraschallkopf 2 zur dentalen Struktur eingestellt werden. Hierdurch kann der Abstand stets nach den konkreten Gegebenheiten

der dentalen Struktur und der genauen Gestaltung der Dentalschiene angepasst werden.

[0065] Zwischen den Stiften 202, 204 und dem Aufnahmeabschnitt 10 kann eine Klemmung vorgesehen sein, derart, dass ein Benutzer nicht unbeabsichtigt den Aufnahmeabschnitt 10 gegenüber den Stiften 202, 204 verschiebt. Ferner kann vorgesehen sein, dass die Stifte 202, 204 nach erfolgter Positionierung in den Bohrungen 206, 208 fixiert werden, beispielsweise mechanisch und/oder stoffschlüssig, etwa durch Verkleben.

ZITATE ENTHALTEN IN DER BESCHREIBUNG

Diese Liste der vom Anmelder aufgeführten Dokumente wurde automatisiert erzeugt und ist ausschließlich zur besseren Information des Lesers aufgenommen. Die Liste ist nicht Bestandteil der deutschen Patent- bzw. Gebrauchsmusteranmeldung. Das DPMA übernimmt keinerlei Haftung für etwaige Fehler oder Auslassungen.

Zitierte Patentliteratur

- DE 199 52 962 A1 [0011]

Zitierte Nicht-Patentliteratur

- Alveolar Bone Segmentation in Intraoral Ultrasonographs with Machine Learning - Nguyen KCT, et al., Journal of Dental Research, May 2020, doi: 10.1177/0022034520920593 [0008]

Patentansprüche

1. Vorrichtung (1) für eine reproduzierbare intraorale Positionierung eines Ultraschallkopfes (2), mit
einer Dentalschiene (4), die einen Dentalschienenkörper (6) aufweist, der an einer intraoralen, anatomischen Struktur (8) temporär positionierbar ist und dessen intraorale Positionierung durch die intraorale, anatomische Struktur (8) definiert ist, wobei der Dentalschienenkörper (6) die intraorale, anatomische Struktur (8) derart nachbildet, dass der Dentalschienenkörper (6) in exakt einer räumlich definierten Position formschlüssig an die intraorale, anatomische Struktur (8) positionierbar ist,
wobei die Dentalschiene (4) einen Aufnahmeabschnitt (10) zum Aufnehmen des Ultraschallkopfes (2) aufweist, wobei der Aufnahmeabschnitt (10) einen Schalldurchtrittsbereich (12) aufweist, durch den Ultraschallwellen von dem Ultraschallkopf (2) zu einem darzustellenden Abschnitt (14) der intraoralen, anatomischen Struktur (8) hindurchtreten können, und wobei der Aufnahmeabschnitt (10) wenigstens ein Positionierelement (16) aufweist, mit dem der Ultraschallkopf (2) in Kontakt gebracht werden kann, um die Pose des Ultraschallkopfes (2) relativ zur Dentalschiene (4) zu definieren.

2. Vorrichtung (1) nach Anspruch 1, wobei das Positionierelement (16) ein Rahmen (18) ist, der zur formschlüssigen Anlage mit dem Ultraschallkopf (2) eingerichtet ist.

3. Vorrichtung (1) nach einem der vorstehenden Ansprüche, wobei der Aufnahmeabschnitt (10) dazu eingerichtet ist, ein mit einem Koppelmittel gefülltes Pad (20) zwischen der intraoralen, anatomischen Struktur (8) und der Vorrichtung (1) in Position zu halten.

4. Vorrichtung (1) nach einem der vorstehenden Ansprüche, wobei der Dentalschienenkörper einen Plattformabschnitt (5) und einen Auflageabschnitt (7) aufweist, wobei der Aufnahmeabschnitt (10) an dem Plattformabschnitt (5) angeordnet ist, und wobei der Auflageabschnitt (7) in exakt einer räumlich definierten Position formschlüssig mit der intraoralen, anatomischen Struktur (8) in Eingriff bringbar ist.

5. Vorrichtung (1) nach Anspruch 4, wobei der Auflageabschnitt (7) durch eine Bohrschablone (22) gebildet ist.

6. Vorrichtung (1) nach Anspruch 5, wobei die Bohrschablone (22) mit dem Plattformabschnitt (5) kraftschlüssig, formschlüssig und/oder stoffschlüssig verbunden ist.

7. Vorrichtung (1) nach einem der vorstehenden Ansprüche 5 oder 6, wobei die Bohrschablone (22) ein erstes Formschlusselement aufweist, und wobei der Plattformabschnitt (5) ein zweites Formschlusselement aufweist, das mit dem ersten Formschlusselement zum formschlüssigen Festlegen der Verbindung von der Bohrschablone (22) und dem Plattformabschnitt korrespondiert.

8. Vorrichtung (1) nach einem der vorstehenden Ansprüche 6 oder 7, wobei der Plattformabschnitt (5) an wenigstens zwei Bohrpositionen (24a, 24b) an der Bohrschablone (22) befestigt ist.

9. Vorrichtung (1) nach einem der vorstehenden Ansprüche 6 bis 8, wobei die Bohrschablone (22) eine Aussparung (26) aufweist, und wobei der Plattformabschnitt (5) formschlüssig in der Aussparung (26) aufgenommen ist.

10. Vorrichtung nach einem der vorstehenden Ansprüche, wobei der Dentalschienenkörper (6) wenigstens einen Stift (202, 204) und der Aufnahmeabschnitt (10) wenigstens eine Bohrung (206, 208) aufweist, durch die sich der Stift (202, 204) erstreckt zum relativen Positionieren des Dentalschienenkörpers (6) und des Aufnahmeabschnitts (10) zueinander.

11. Verfahren (100) zum Herstellen einer Vorrichtung für die reproduzierbare intraorale Positionierung eines Ultraschallkopfes, umfassend:
- Bereitstellen einer Bohrschablone, die in exakt einer räumlich definierten Position formschlüssig mit einer intraoralen, anatomischen Struktur in Eingriff bringbar ist, und die eine Bohrführung aufweist zur Führung eines Bohrers zum Fertigen einer Bohrung für ein Implantat in einer intraoralen, anatomischen Struktur (102);
- Bereitstellen eines Plattformabschnitts, an dem ein Aufnahmeabschnitt zum Aufnehmen eines Ultraschallkopfes angeordnet ist, wobei der Aufnahmeabschnitt einen Schalldurchtrittsbereich aufweist, durch den Ultraschallwellen hindurchtreten können, und wobei der Aufnahmeabschnitt wenigstens ein Positionierelement aufweist, mit dem der Ultraschallkopf in Kontakt gebracht werden kann (104);
- Verbinden der Bohrschablone mit dem Plattformabschnitt, wobei bei der Bohrschablone ein Kronenabschnitt entfernt wird, wobei der Kronenabschnitt ein Abschnitt der Bohrschablone ist, der bei intraoraler Positionierung an und/oder auf einer Krone eines in der intraoralen, anatomischen Struktur eingesetzten Implantats liegen würde, und wobei der Kronenabschnitt die Bohrführung umfasst (106).

12. Verwendung einer Vorrichtung für eine intraorale Positionierung eines Ultraschallkopfes, vorzugsweise nach einem der vorstehenden Ansprüche 1 bis 10, zum Aufnehmen von intraoralen Ultra-

schallbilden eines darzustellenden Abschnitts einer intraoralen, anatomischen Struktur mit einem Ultraschallkopf.

Es folgen 9 Seiten Zeichnungen

Anhängende Zeichnungen

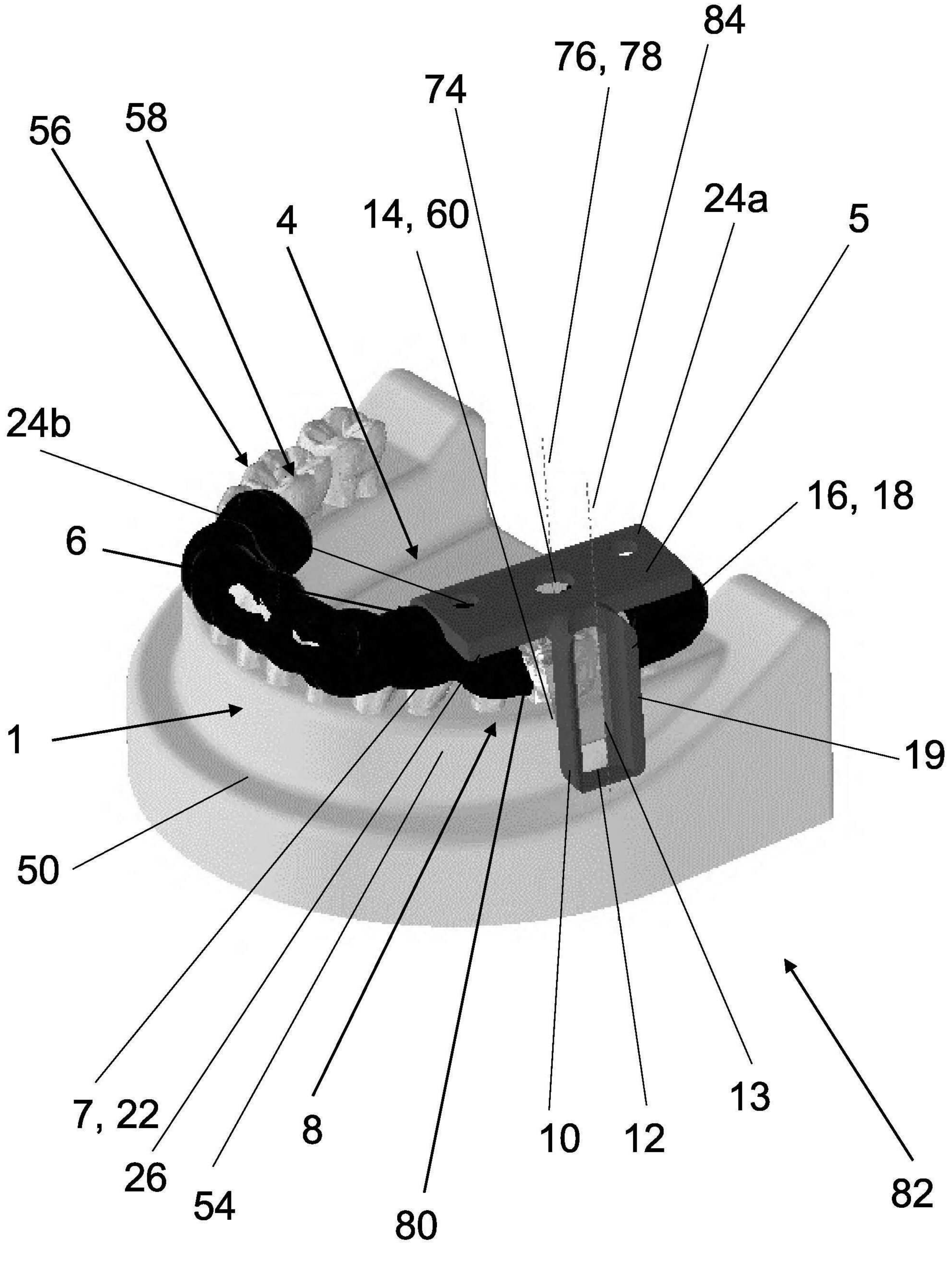

Fig. 1

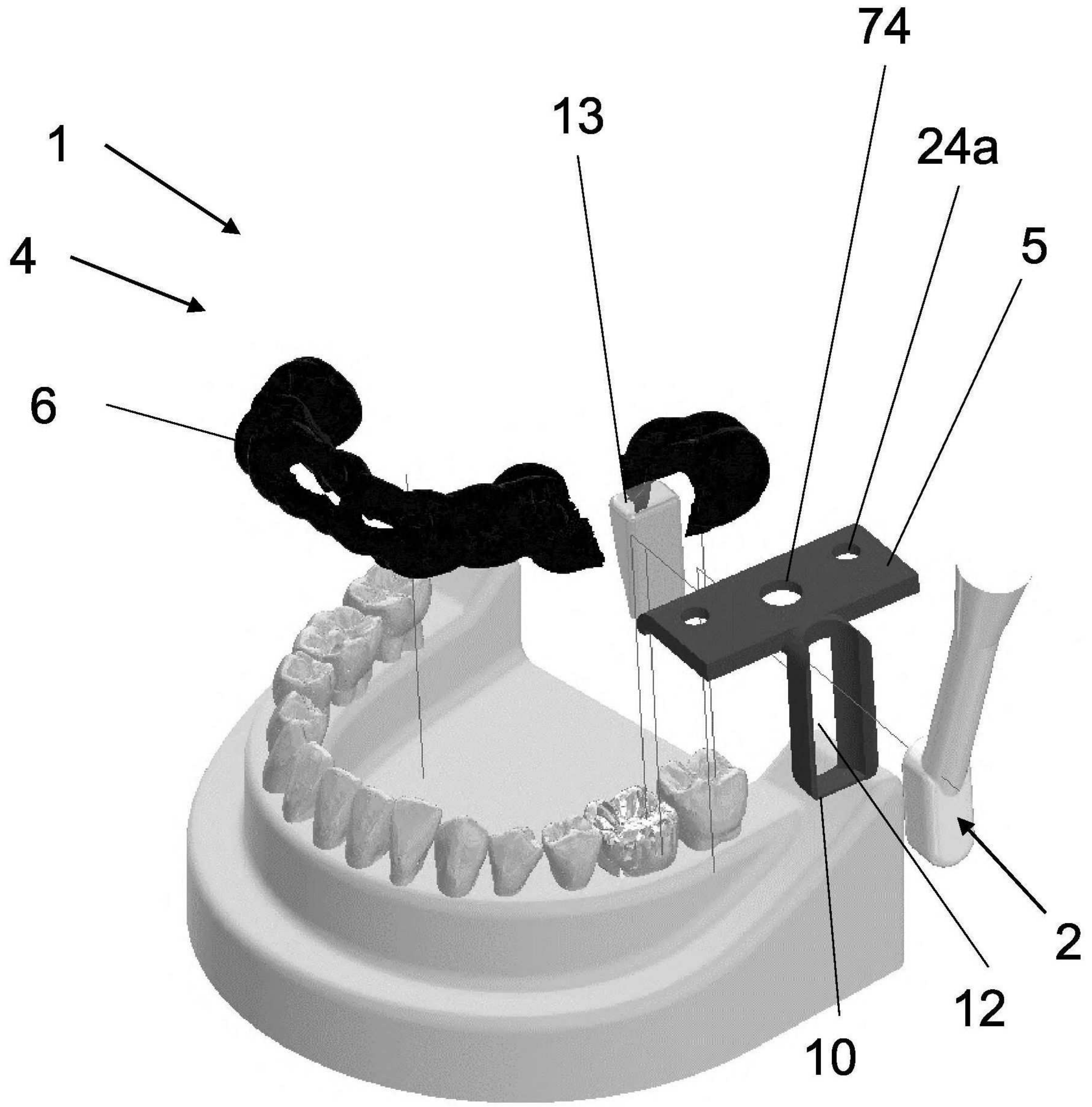

Fig. 2

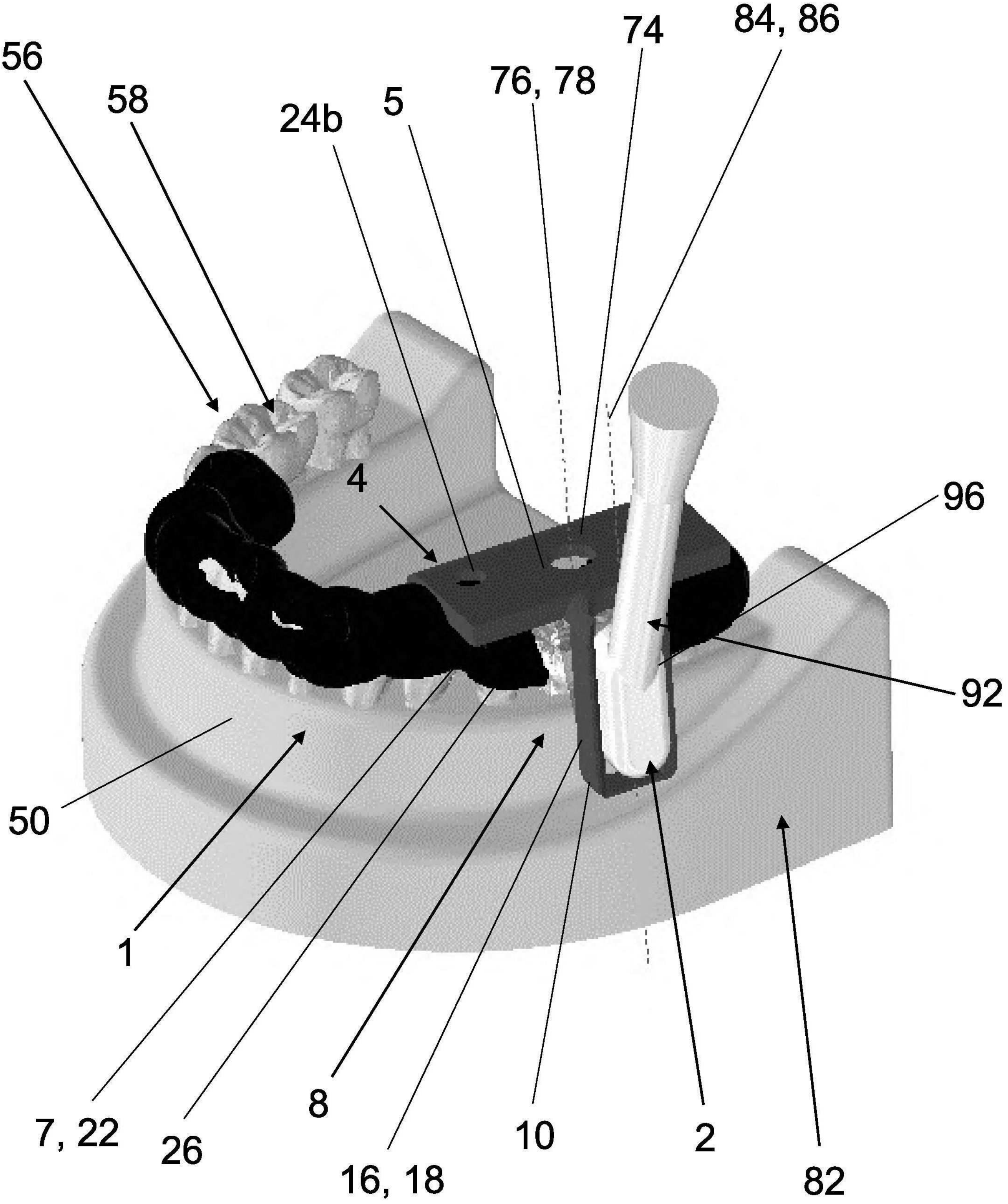

Fig. 3

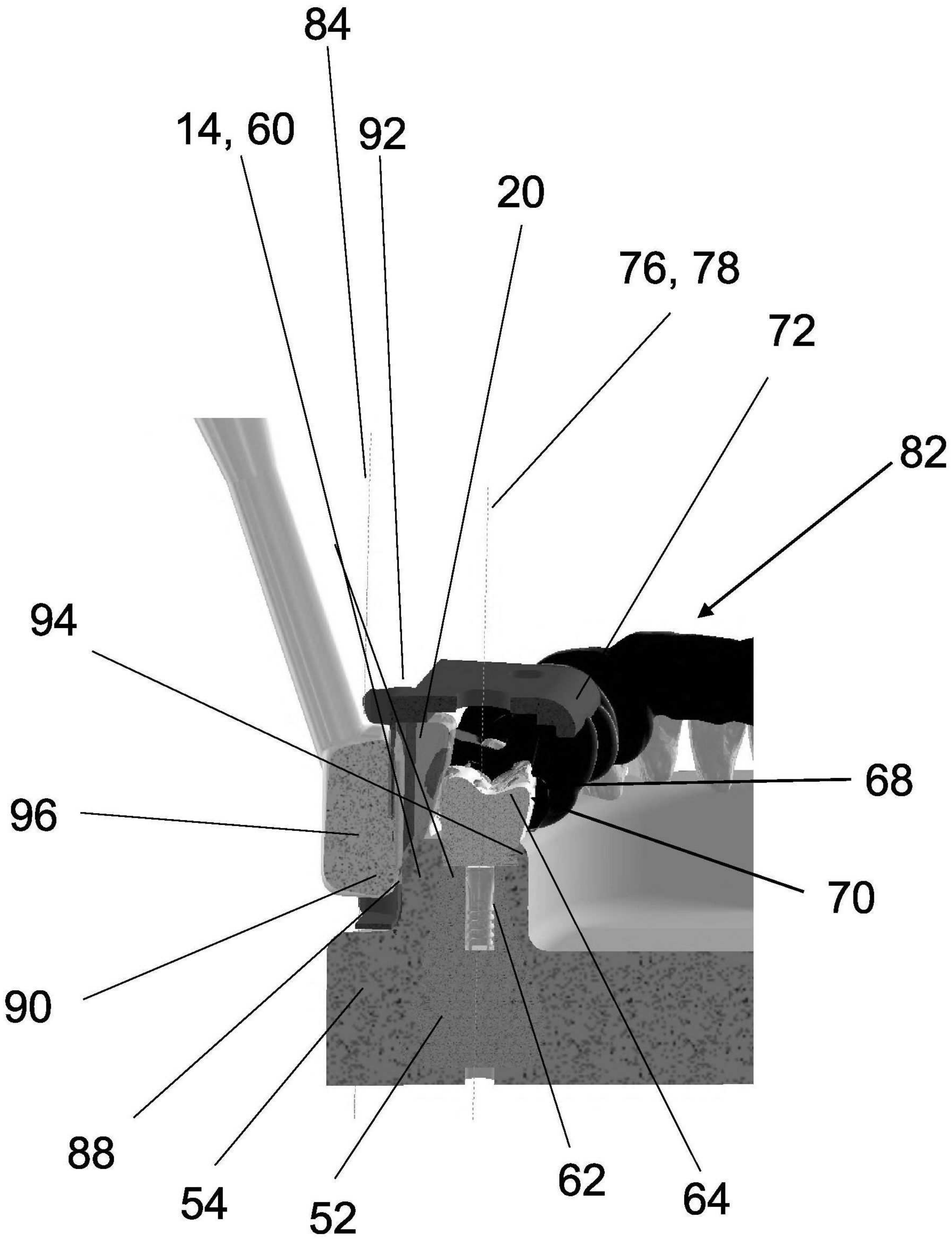

Fig. 4

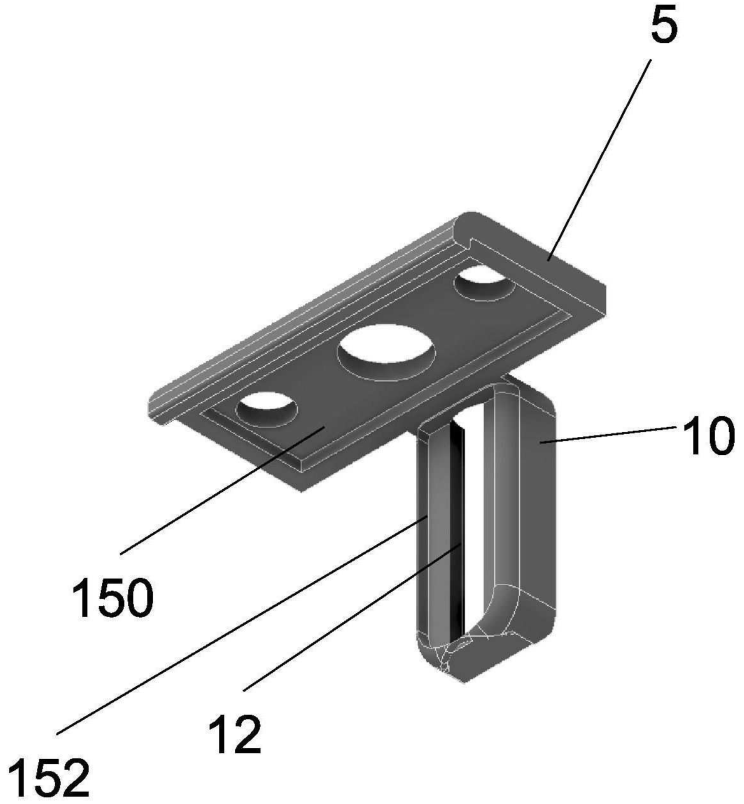

Fig. 5a

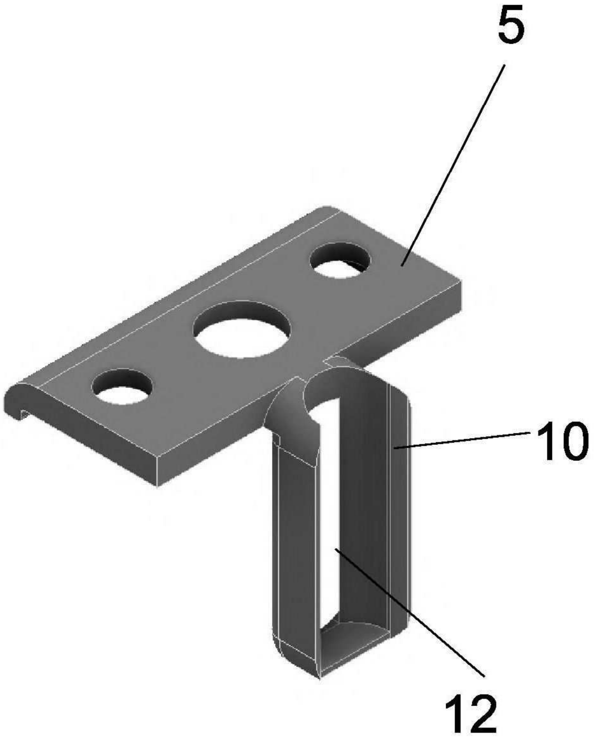

Fig. 5b

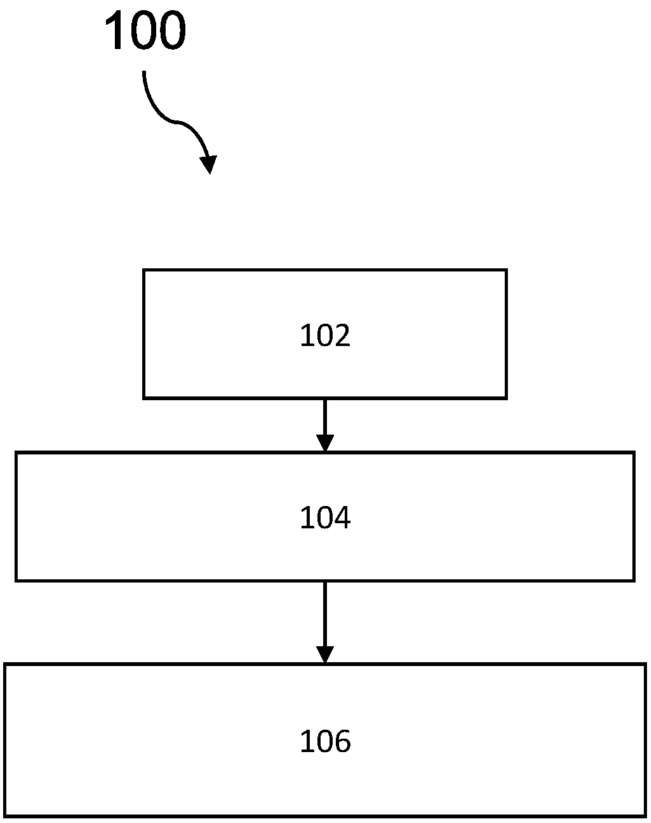

Fig. 6

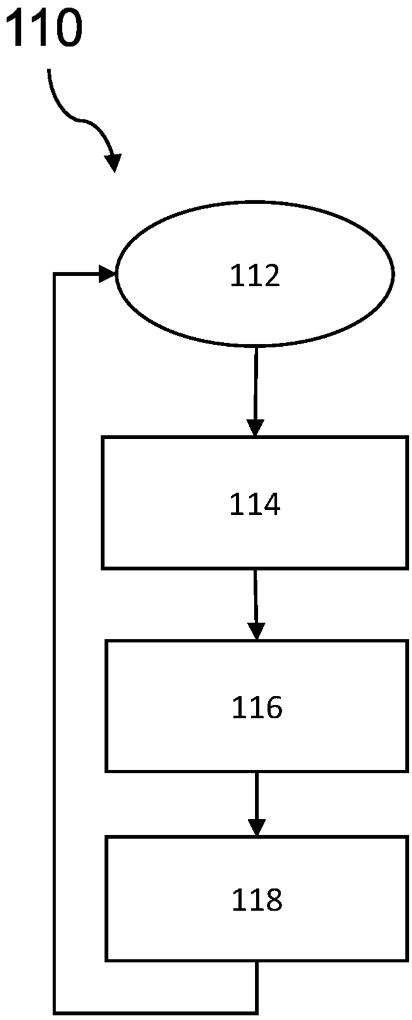

Fig. 7

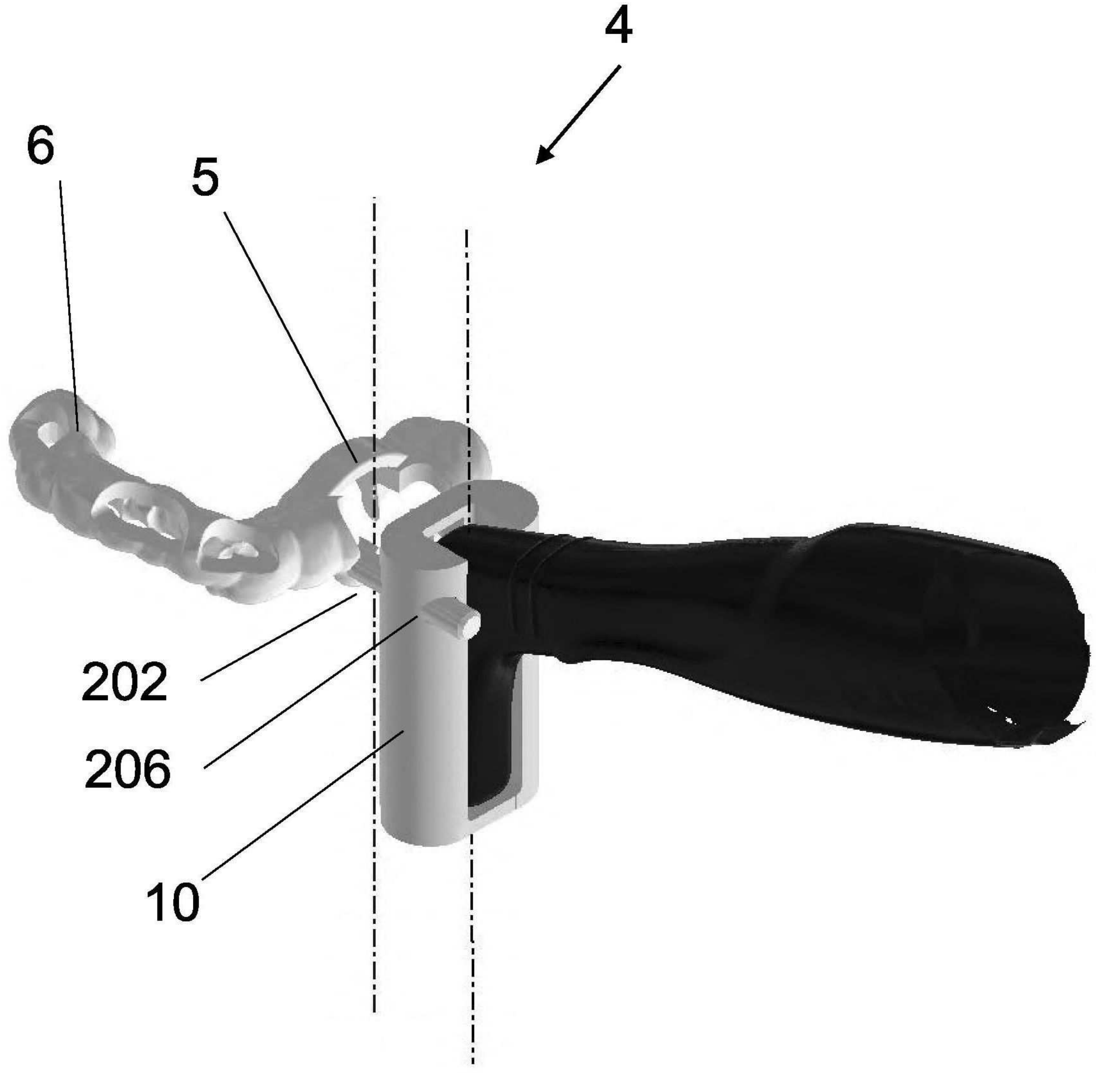

Fig. 8

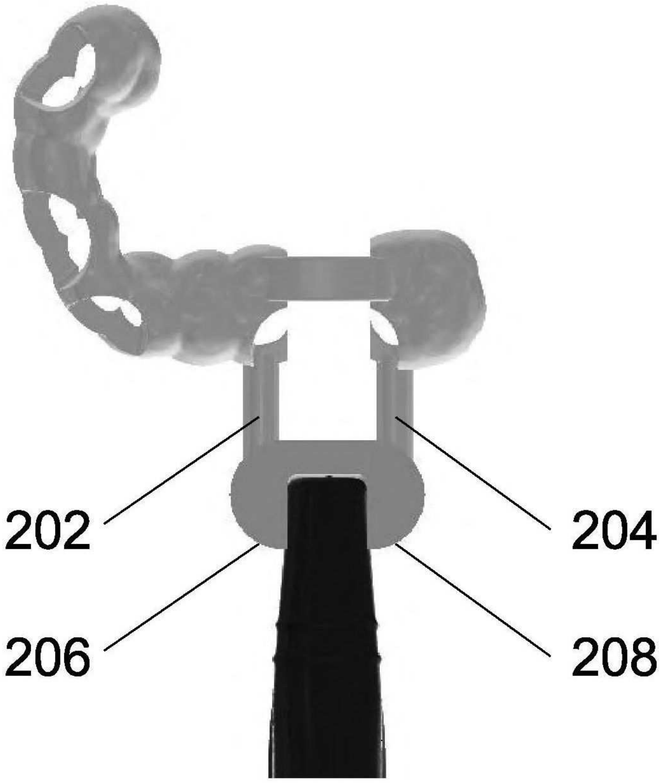

Fig. 9

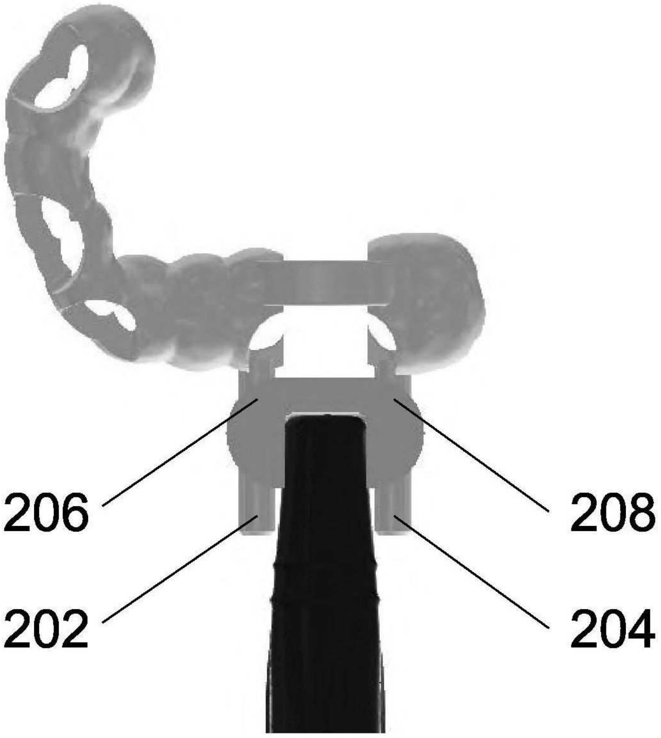

Fig. 10

 Das Dokument wurde durch die Firma Luminess hergestellt.
