# Supplementary material for: Ultrasound-based assessment of peri-implant mucosal thickness: an ex vivo comparative study with artificial intelligence-assisted image analysis
Source: BMC Oral Health. 2026 Jun 27;26:1215. doi: 10.1186/s12903-026-08665-0 (PMC13343591; doi:10.1186/s12903-026-08665-0)

Additional file 2: Q-Q Plots of Residuals from the Linear Mixed Model (with and without Log Transformation)

Q-Q plots were generated to evaluate the assumption of normality of residuals from the linear mixed-effects model (LMM). Residuals were assessed both for the raw (untransformed) data and for the log-transformed data. For each dataset, standard Q-Q plots as well as detrended Q-Q plots were created. Visual inspection indicated that log transformation improved the adherence of residuals to the assumption of normality, justifying its use in the final LMM. All plots were generated using a statistical software package (SPSS Statistics v29.0.2.0; IBM Corp., Armonk, NY, USA).

a. Q-Q Plot of Residuals (Log Transformation)

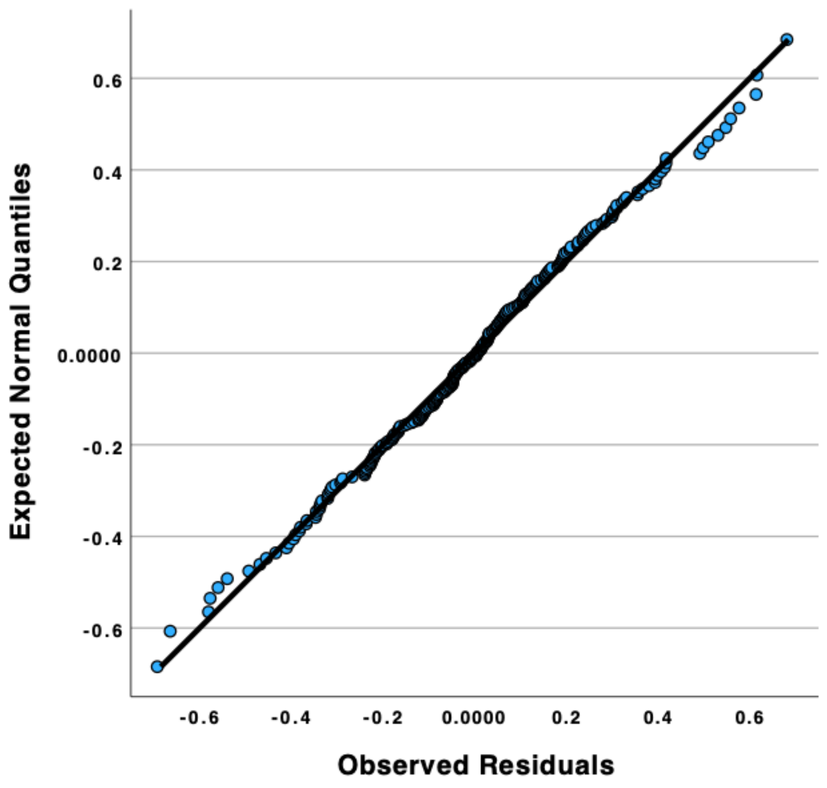

b. Q-Q Plot of Residuals (Raw Data without Log Transformation)

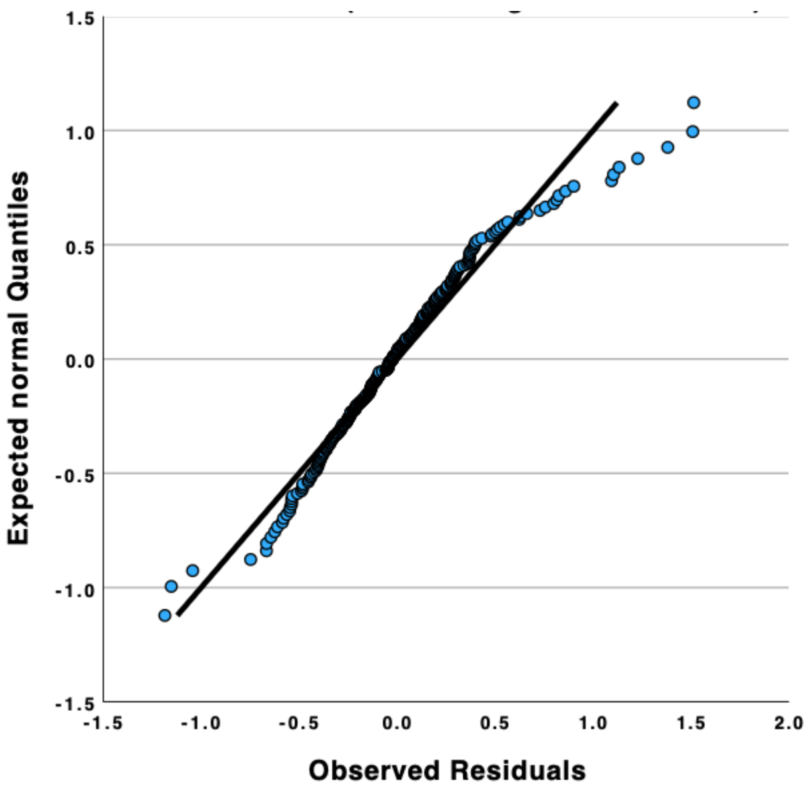

c. Detrended Q-Q Plot of Residuals (Raw Data without Log Transformation)

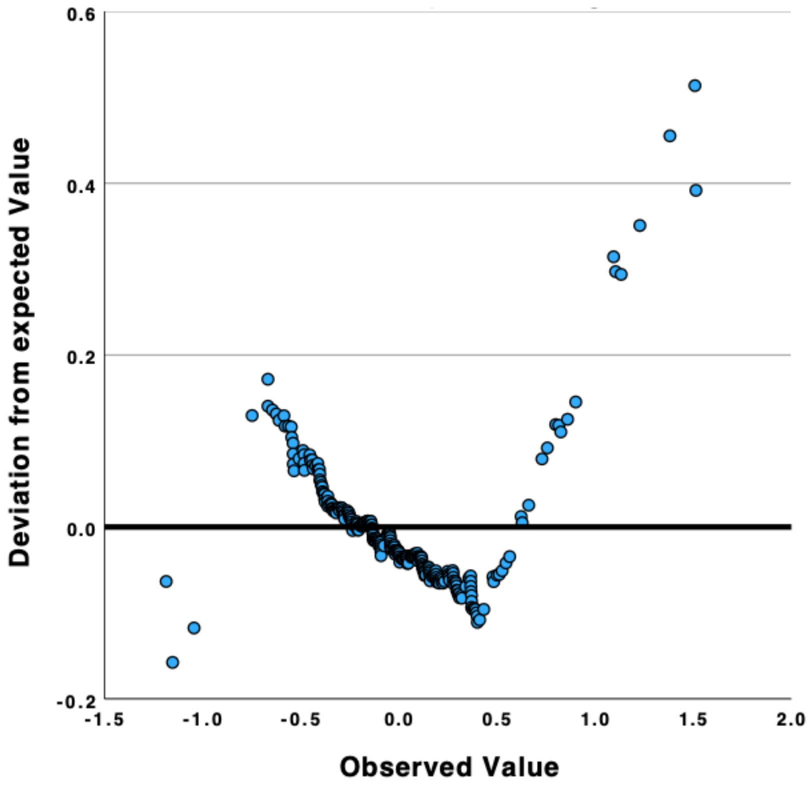

d. Detrended Q-Q Plot of Residuals (Log Transformation)

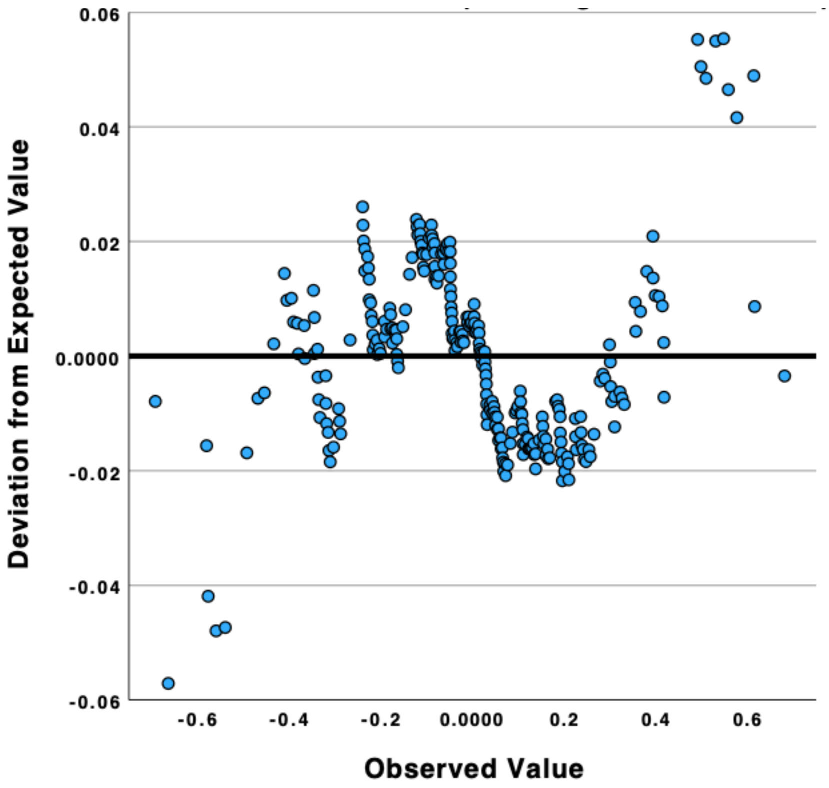

Supplement: Supplementary file 2 — Additional file 2: LMM Q-Q-Plot of Residuals Log-Data and Raw-Data. [file 12903_2026_8665_MOESM2_ESM.pdf]
