# Supplementary material for: Ultrasound-based assessment of peri-implant mucosal thickness: an ex vivo comparative study with artificial intelligence-assisted image analysis
Source: BMC Oral Health. 2026 Jun 27;26:1215. doi: 10.1186/s12903-026-08665-0 (PMC13343591; doi:10.1186/s12903-026-08665-0)

Additional file 3: Scatterplots of Residuals for Testing Homoscedasticity in the Linear Mixed-Effects Model (Log-Transformed Data)

To evaluate the assumptions of the linear mixed-effects model (LMM), scatterplots of residuals were generated using the statistical software package (SPSS Statistics v29.0.2.0; IBM Corp., Armonk, NY, USA). These plots assess the assumption of homoscedasticity, i.e., whether the residuals show constant variance across the range of predicted values. Both scatterplots are presented: one plotting residuals against the total predicted values (fixed and random effects), and another plotting residuals against fixed-effects predictions only. A random distribution of residuals without systematic patterns supports the validity of the homoscedasticity assumption.

a. Scatterplot of residuals versus total predicted values (fixed + random effects).

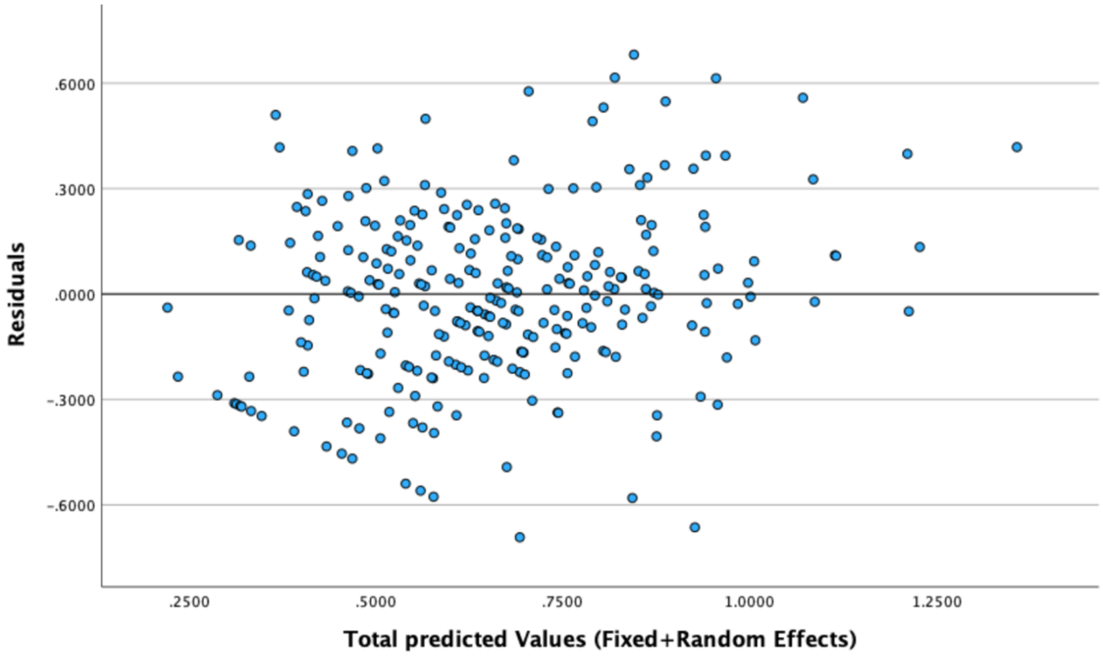

b. Scatterplot of residuals versus fixed-effects predicted values.

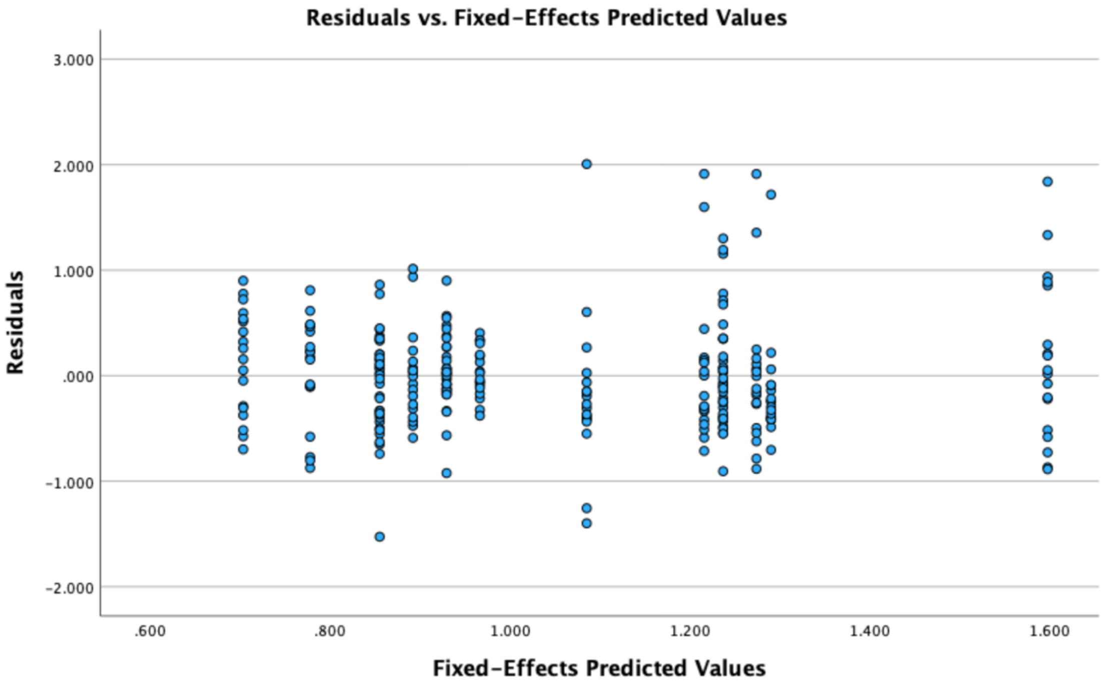

Supplement: Supplementary file 3 — Additional file 3: LMM Scatterplots Homoscedasticity Log-Data. [file 12903_2026_8665_MOESM3_ESM.pdf]
