# Supplementary material for: Ultrasound-based assessment of peri-implant mucosal thickness: an ex vivo comparative study with artificial intelligence-assisted image analysis
Source: BMC Oral Health. 2026 Jun 27;26:1215. doi: 10.1186/s12903-026-08665-0 (PMC13343591; doi:10.1186/s12903-026-08665-0)

Additional file 4: Histograms of residuals and Q-Q plots

Residual histograms and Q-Q plots were generated using a statistical software package (SPSS Statistics v29.0.2.0; IBM Corp., Armonk, NY, USA) to assess the assumption of normally distributed residuals. This assumption is required for valid interpretation of Bland–Altman analyses and intraclass correlation coefficients (ICC). In the histograms, a symmetric, bell-shaped distribution of residuals indicates approximate normality. In the Q-Q plots, points closely following the diagonal line reflect good agreement between observed and theoretical quantiles, supporting the assumption of normality.

a. Residual histogram for expert-annotated ultrasound (US) versus transgingival probing (TP)

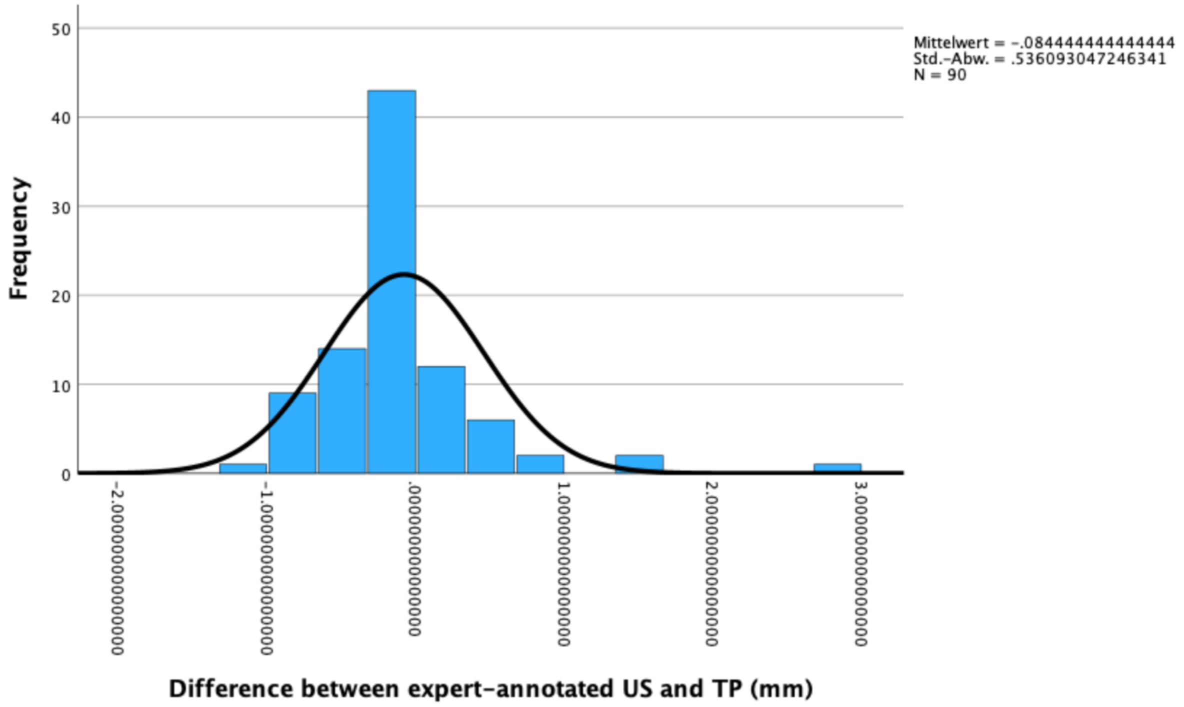

b. Residual histogram for expert-annotated US versus AI-segmented US

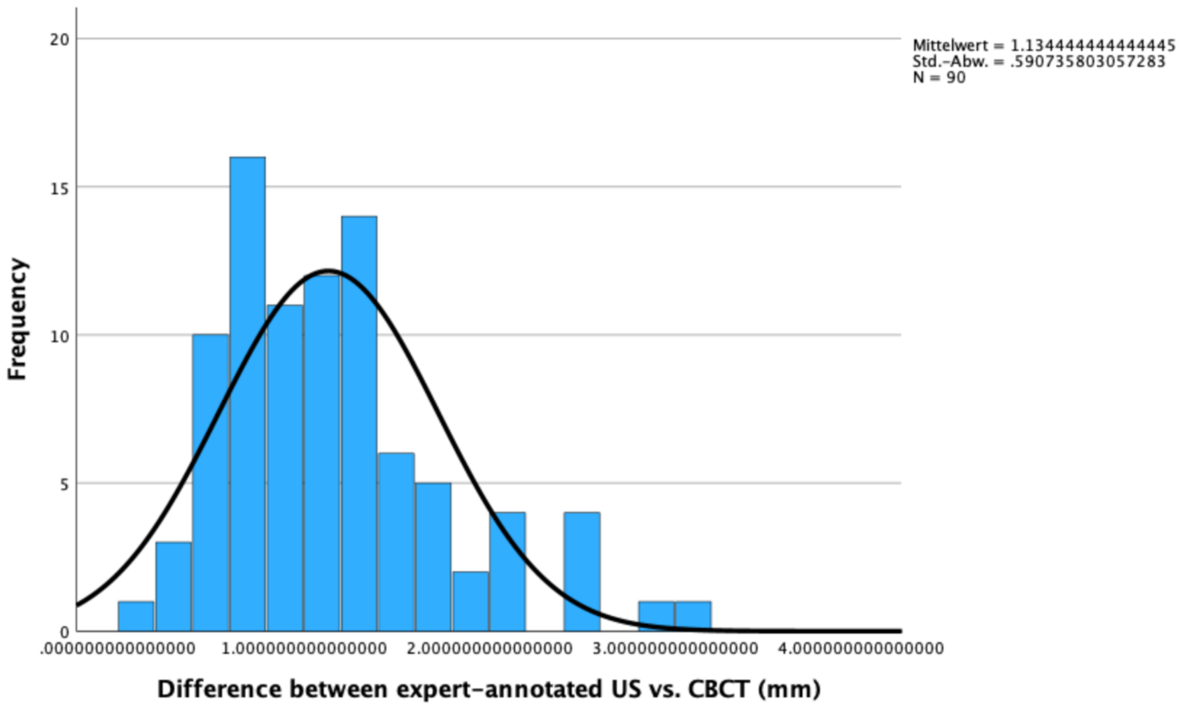

c. Residual histogram for expert-annotated US versus CBCT

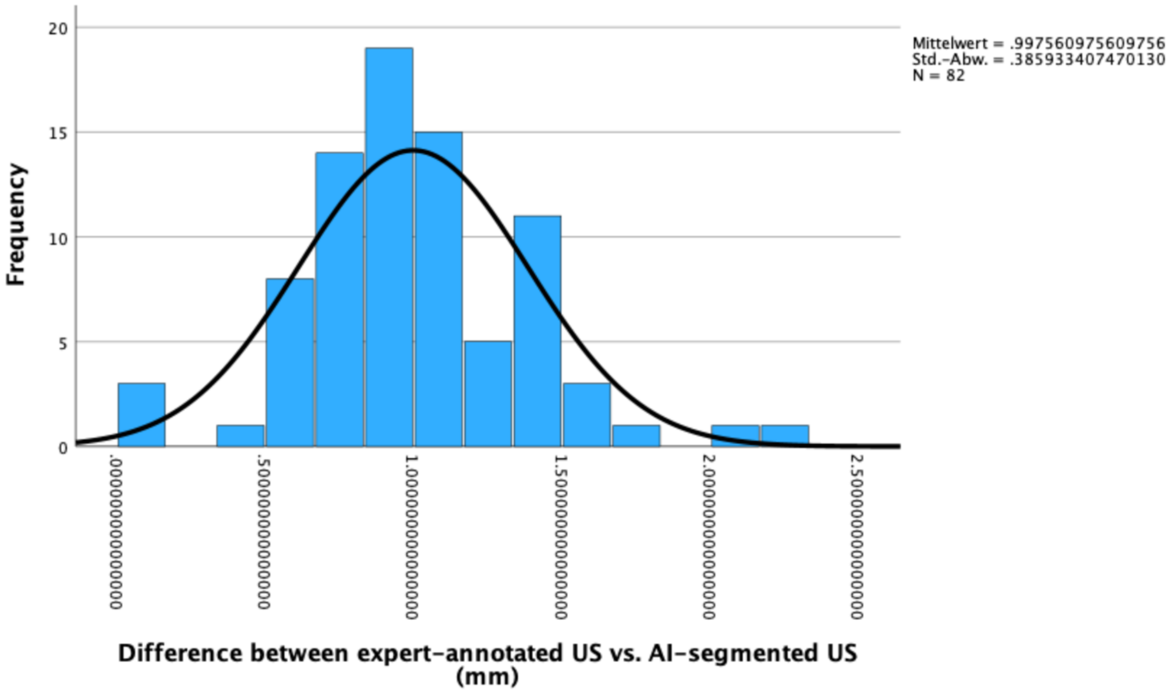

d. Q-Q Plot for expert-annotated US versus TP

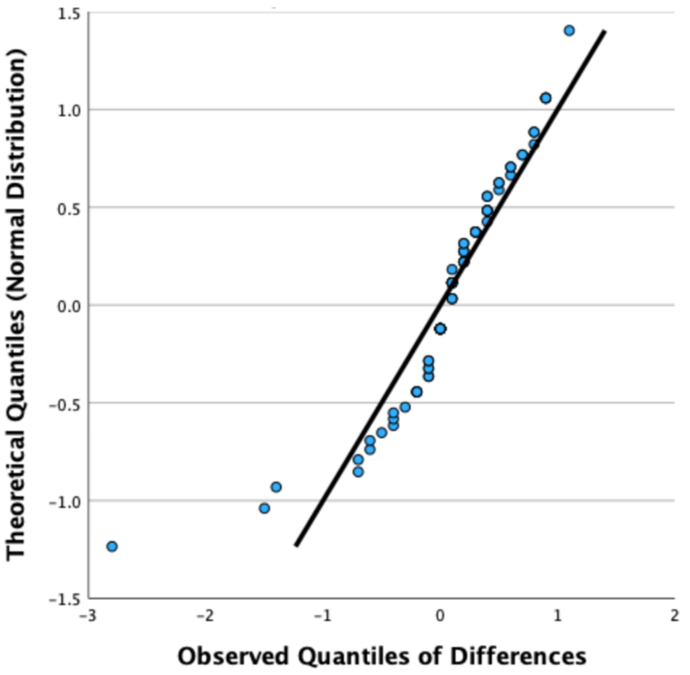

e. Q-Q plot for CBCT versus TP

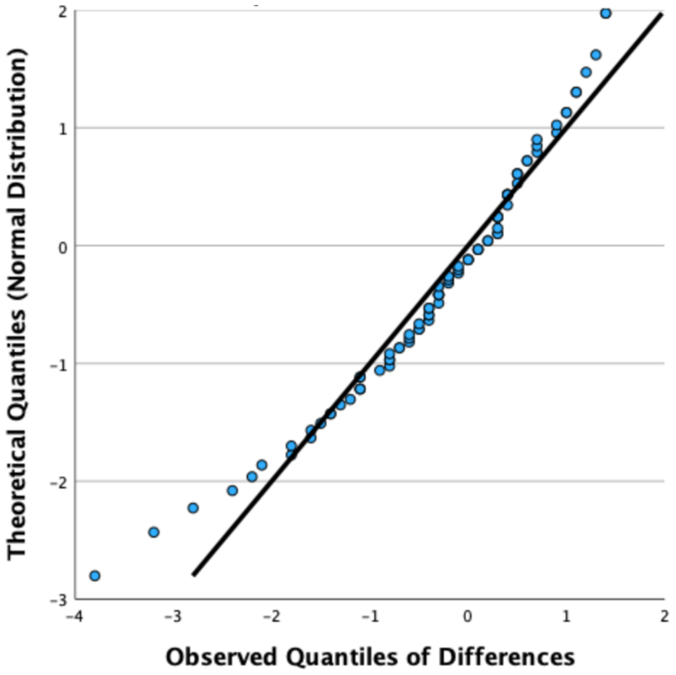

f. Q-Q plot for expert-annotated US versus AI-segmented US

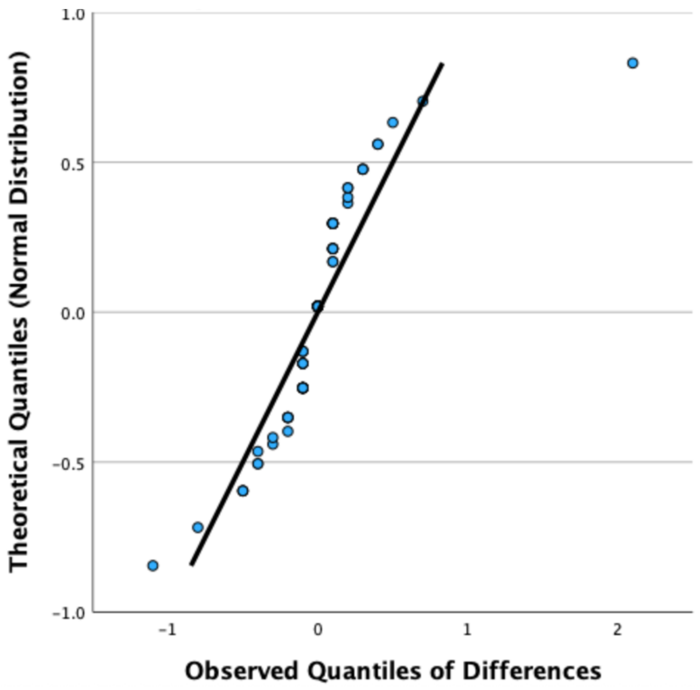

Supplement: Supplementary file 4 — Additional file 4: Histograms of Residuals Q-Q-Plots. [file 12903_2026_8665_MOESM4_ESM.pdf]
